# Comparative Analyses of Chloroplast Genomes of Cucurbitaceae Species: Lights into Selective Pressures and Phylogenetic Relationships

**DOI:** 10.3390/molecules23092165

**Published:** 2018-08-28

**Authors:** Xiao Zhang, Tao Zhou, Jia Yang, Jingjing Sun, Miaomiao Ju, Yuemei Zhao, Guifang Zhao

**Affiliations:** 1 Key Laboratory of Resource Biology and Biotechnology in Western China (Ministry of Education), College of Life Sciences, Northwest University, Xi’an 710069, China; zhxiaao@163.com (X.Z.); yjhgxd@stumail.nwu.edu.cn (J.Y.); sjjnwu@163.com (J.S.); jumm089@163.com (M.J.); 2 School of Pharmacy, Xi’an Jiaotong University, Xi’an 710061, China; zhoutao196@mail.xjtu.edu.cn; 3 College of Biopharmaceutical and Food Engineering, Shangluo University, Shangluo 726000, China; yezi19820320@163.com

**Keywords:** Cucurbitaceae, chloroplast genome, structural comparison, selective pressures, RNA editing, phylogeny

## Abstract

Cucurbitaceae is the fourth most important economic plant family with creeping herbaceous species mainly distributed in tropical and subtropical regions. Here, we described and compared the complete chloroplast genome sequences of ten representative species from Cucurbitaceae. The lengths of the ten complete chloroplast genomes ranged from 155,293 bp (*C. sativus*) to 158,844 bp (*M. charantia*), and they shared the most common genomic features. 618 repeats of three categories and 813 microsatellites were found. Sequence divergence analysis showed that the coding and IR regions were highly conserved. Three protein-coding genes (*accD*, *clpP*, and *matK*) were under selection and their coding proteins often have functions in chloroplast protein synthesis, gene transcription, energy transformation, and plant development. An unconventional translation initiation codon of *psbL* gene was found and provided evidence for RNA editing. Applying BI and ML methods, phylogenetic analysis strongly supported the position of *Gomphogyne*, *Hemsleya*, and *Gynostemma* as the relatively original lineage in Cucurbitaceae. This study suggested that the complete chloroplast genome sequences were useful for phylogenetic studies. It would also determine potential molecular markers and candidate DNA barcodes for coming studies and enrich the valuable complete chloroplast genome resources of Cucurbitaceae.

## 1. Introduction

As the fourth most economically important plant family, Cucurbitaceae consists of 115 proposed genera with approximately 960 species distributed in tropical and subtropical areas [1]. The vast majority of these plants are annual vines and woody lianas, and only a small proportion are shrubs and trees [2]. Cultivars developed by breeders, especially melon (*Cucumis melo*), watermelon (*Citrullus lanatus*), bottle gourd (*Lagenaria siceraria*), pumpkin (*Cucurbita pepo*), and cucumber (*Cucumis sativus*), are the basis for industries [3]. Their fruits are not only edible, but also used by humans mostly as durable containers, fishnet floats, and musical instruments [4]. The commercial use of derivatives from medicinal species is increasing rapidly. For instance, flavonoids and saponins contained in *Gynostemma pentaphyllum* [5] have radical scavenging and antiproliferative properties [6], and cucurbitane-type compounds extracted from *Hemsleya amabilis* and *Hemsleya carnosiflora* exert anti-inflammatory functions in bronchitis and tuberculosis treatments [7,8,9]. These plants are also used as traditional Chinese medicinal herbs because of their anticancer effect [6,10]. Therefore, over the last decades, large amounts of research have paid much attention to the improvement of cultivated varieties and the development of medicinal value. However, the most important basis of developing natural medicine is the wild species’ identification, which is also very difficult. Take the genera *Gomphogyne*, *Hemsleya*, and *Gynostemma* as examples: they are all morphologically creeping and herbaceous with 3-11-foliolate leaves in above-ground plants. Although *Gomphogyne* ismonoecious, *Gynostemma* is dioecious, and *Hemsleya* has enlarged underground tubers, it has been rather problematic to define the classification of these species in the wild, especially without flowering or excavation [11].

In addition, the existing studies about Cucurbitaceae mainly focus on the history of domestication, origin, and dispersal [1,12,13,14,15]. Nevertheless, the phylogeny of Cucurbitaceae family has not yet been clearly solved. Due to the description and validation of new species, the number of interspecies and the attribution problem of some genera remain uncertain, such as with *Hemsleya* and *Gomphogyne.* Although some molecular-based phylogenic studies have been carried out on many Cucurbitaceae genera, *Hemsleya* and *Gomphogyne* were either not involved [16,17,18] or just participated in systematic surveys based on some specific fragments of DNA with a limited number of species within each genus [19,20]. Therefore, we prefer using the whole complete chloroplast genomes (CPGs) to resolve the phylogenic problem of the genera in the Cucurbitaceae family. Meanwhile, more DNA barcodes from genomic resources are in demand, for use in the identification of species among genera in the Cucurbitaceae family, and in further studies to reveal the genetic diversity, population structure, origin, and evolution of these species.

Comparatively speaking, genome-wide datasets have an edge over traditional DNA markers in providing information to effectively solve historically complex phylogenetic relationships [21,22,23]. The chloroplast genome (CPG) is a circular double-stranded DNA molecule which has maternal inheritance in the majority of plants [24,25]. It is smaller than the nuclear genome in size, and has a moderate rate of nucleotide evolution, but shows a difference in the rate of divergence between protein coding (CDS) and noncoding (CNS) regions [26]. Previous studies have demonstrated that most CPGs of angiosperms have a stable quadripartite structure: a pair of inverted repeats regions (IRa and IRb), one large single-copy region (LSC) and one small single-copy region (SSC) [24]. The common sizes of CPGs range from 120 kb to 160 kb usually caused by contractions and expansions of IR regions [27]. The comparative analyses of complete CPGs could contribute to understanding the complete CPG structure and evolution, identification of species and phylogenetic relationships [28].

In this study, comparative analyses were applied on complete CPGs of ten representative species in Cucurbitaceae to explore structural differentiation and molecular evolution of CPG sequences and increase the number of valuable complete CPG resources. The characterization of highly variable regions would contribute to developing candidate DNA barcodes for future studies. Microsatellites (SSRs) could be used as potential molecular polymorphic markers to reveal the genetic diversity and population structure of Cucurbitaceae. The identification of protein-coding genes under selection would play an important role in the analyses of adaptive evolution for plants in ecosystems. Furthermore, this study would reconstruct the intergeneric relationships and locate the phylogenetic position of genera *Gomphogyne* and *Hemsleya* in Cucurbitaceae.

## 2. Results

### 2.1. Genome Features

**Figure 1:**
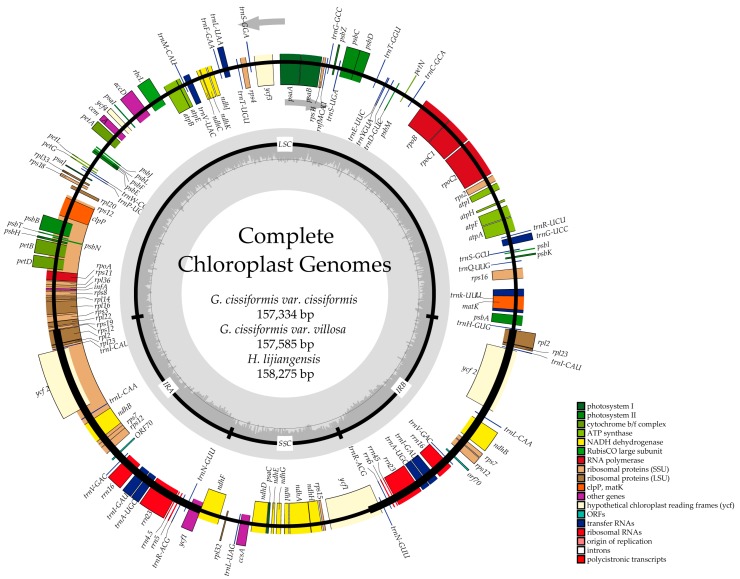
Gene maps of chloroplast genomes of Cucurbitaceae. Genes on the inside of the large circle are transcribed clockwise and those on the outside are transcribed counterclockwise. The genes are color-coded based on their functions. Dashed area represents the GC composition of the chloroplast genome.

For three newly-obtained CPGs, the mean coverage of raw reads ranged from 1112.4 to 1392.7 (Table 1), and the lengths of consensus sequences were 157,334 bp (*G. cissiformis* var. *cissiformis*), 156,585 bp (*G. cissiformis* var. *villosa*) and 158,275 bp (*H. lijiangensis*). Each of them encoded 133 genes including 87 protein-coding genes, eight rRNA genes, 37 tRNA genes, and one pseudogene (Table 1 and Table 2; Figure 1).

**Figure 2:**
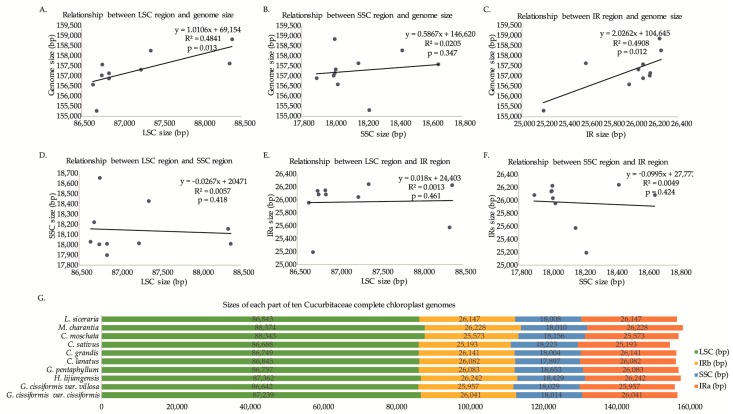
Relationships between complete chloroplast genome sizes and LSC, SSC and IR regions lengths, respectively. (**A**–**F**) Correlational relationships among each region; (**G**) sizes of each part of ten Cucurbitaceae complete chloroplast genomes.

The comparative analyses of whole CPGs from ten species of Cucurbitaceae showed that the sizes of 10 CPGs ranged from 155,293 bp (*C. sativus*) to 158,844 bp (*M. charantia*), with an average CPG sequence length of 157,264 bp. All of the CPGs displayed a typical quadripartite structure: an LSC region (ranged from 86,642 bp to 88,374 bp) and an SSC region (ranged from 17,897 bp to 18,653 bp) which were separated by two IR regions (ranged from 25,193 bp to 26,242 bp; Table 1, Figure 2). The LSC region and IR region had a significant correlational relationship with the overall genome size, and each of the structural regions of the CPGs were not correlated with each other (Figure 2). A comparison of CPG sequences among ten species showed that there was no dramatic difference in compared features. The GC content percentage of *C. lanatus* (37.2%) was more than any of the other genomes (36.7–37.1%), while *M. charantia* had the lowest GC content (36.7%). For four structural regions, the GC content of IR region (42.7–43.1%) was clearly higher than that of the LSC (34.3–34.9%) region and SSC (30.6–31.8%) region for each CPG (Table 1). The CPGs encoded 122 to 135 functional genes including some pseudogenes, i.e., the *infA* gene in *G. cissiformis* var. *cissiformis*, *G. cissiformis* var. *villosa*, *H. lijiangensis*, and *G. pentaphyllum*, the *rps16* gene in *C. sativus*, and the *ycf1* gene in *M. charantia* and *L. siceraria* (Table 1 and Table 2).

### 2.2. IR/SC Boundary, Genome Rearrangement and Sequence Divergence

**Figure 3:**
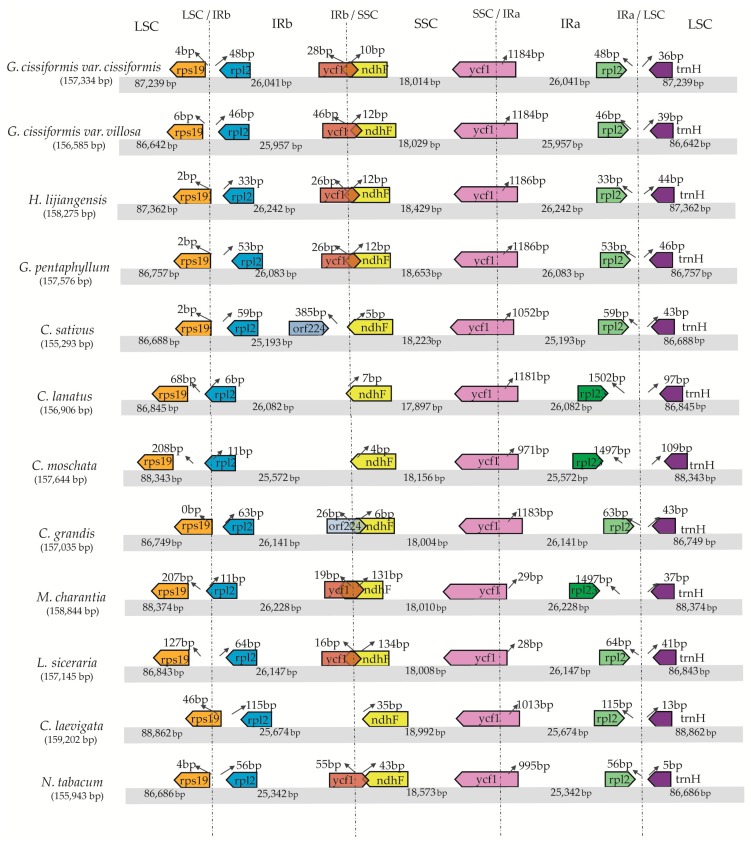
Comparison of the LSC, IR, and SSC border regions among the 10 Cucurbitaceae chloroplast genomes. Number above the gene features means the distance between the ends of genes and the borders sites. These features are not to scale.

The IR/SC boundary areas of 10 CPGs of Cucurbitaceae species and two outgroups were compared (Figure 3). The gene content and order were observed to have some differences, for example, gene *ycf1* and gene *rpl2* were lost in two LSC borders of *M. charantia* and *L. siceraria*; gene *orf224* existed in the IRb border of *C. sativus* and *C. grandis*; as well as the location of gene *rps19* was diversified in all of the examined species (Figure 3). The expansions and contractions of IR region were discovered. Taking *M. charantia* as an example, gene *rps19* that was located in the LSC region was 207 bp away from the LSC/IRb boundary, while this distance was 0–6 bp in some other species, and gene *rpl12* was located in the IRb region, straddling the LSC/IRb border. Gene *ycf1*, located in IRb region, had 131 bp beyond the IRb/SSC boundary, and the comparable region was 10–12 bp long in some other species. This indicated that the relative position of the LSC/IRb boundary had moved backwards, and the IRb/SSC boundary, forwards. Correspondingly, the *ycf1* gene, located in SSC region, had just 29 bp across the SSC/IRa boundary, while this similar region was 971–1186 bp in most of the other species. Both of these phenomena demonstrated a contraction of two IR regions in the complete CPGs.

**Figure 4:**
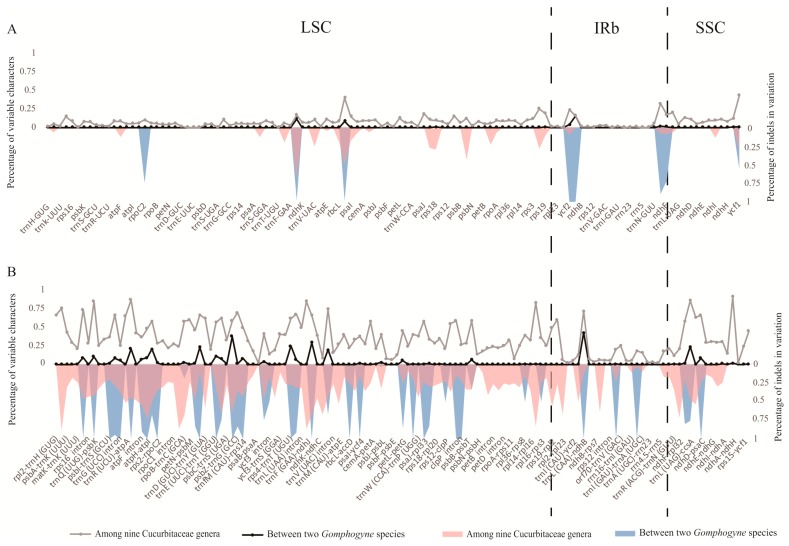
Percentage of variable characters in aligned Cucurbitaceae chloroplast genomes. (**A**) Coding region (CDS) and (**B**) Noncoding region (CNS). These regions are oriented according to their locations in the chloroplast genome.

The whole-genome alignment of the 10 CPGs showed that there were no rearrangement events in Cucurbitaceae (Figure S1). Using *G. cissiformis* var. *cissiformis* as the reference, the alignment of 11 Cucurbitales CPGs were performed to investigate the level of sequence divergence (Figure S2). The result showed a high sequence similarity within genus *Gomphogyne*, but great divergence among different genera and families. As expected, the SC and CNS regions exhibit more differences than IRs and CDS regions, respectively. Moreover, the percentage of variable features in coding and non-coding regions, and the percentage of indels in each variation, were calculated based on two patterns of sequence alignment: (1) two species within *Gomphogyne*; and (2) the represented species of nine genera within Cucurbitaceae. The results showed that the percentage of variable features of CNS ranged from 0 to 43.00% for two *Gomphogyne* species and from 0 to 89.25% for nine Cucurbitaceae genera, with an average level of 2.92% and 33.49% respectively. These percentages were much higher than those of the CDS: from 0 to 16.05% for two *Gomphogyne* species, and from 0 to 42.64% for nine Cucurbitaceae genera, with an average level of 0.41% and 7.49%, respectively (Figure 4, Table S3). This also indicated that CDS was much more conservative than CNS. Mostly, the variations were located in SC regions instead of IR regions. The results also suggested that the variations among nine genera were higher than those between species within a single genus, and most variations were caused by indels (Figure 4). In addition, the top- four highly-variable genes (*accD*, *rpl22*, *ycf1*, and *ycf1*) and top-four highly divergent intergenic regions (*trnR (UCU)-atpA*, *trnL (UAA)-trnF (GAA)*, *rpl32-trnL (UAG)*, and *ndhA intron*) were confirmed (Table S3) and primers for these regions were shown in Supplementary Table S4. These regions could be used as candidate DNA fragments for further studies related to genetics, phylogeny, and species identification.

### 2.3. Repeat Analysis and Microsatellites (SSR)

**Figure 5:**
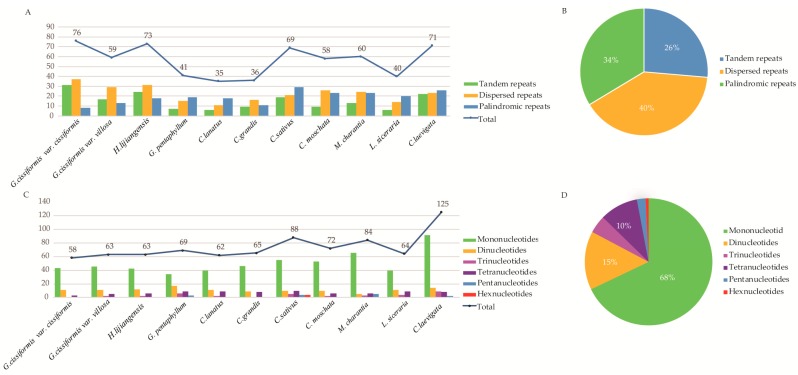
The type and presence of repeated sequences and simple sequence repeats (SSR) in the chloroplast genomes of eleven Cucurbitales species. (**A**) Number of three-types repeats; (**B**)Percentage of three repeat types; (**C**) Number of SSRs and their types; (**D**) Percentage of SSR types.

Three categories of repeats (tandem, dispersed, and palindromic repeats) were identified in the 11 Cucurbitales CPGs (Figure 5A, Tables S5, S6, and S8). A total of 618 repeats were identified for these species including 163 tandem repeats, 247 dispersed repeats, and 208 palindromic repeats, indicating the highest percentage (40%) of dispersed repeats (Figure 5B). Among different species, the number of repeats for *G. cissiformis* var. *cissiformis* (76) and *C. grandis* (36) were the most and the least, respectively (Figure 5A). Additionally, 813 SSRs were found, of which, the number of mono-, di-, tri-, tetra-, penta-, and hexanucleotide repeats were 552, 121, 37, 79, 18, and 6, respectively. It was shown that the mononucleotide repeats were most common, accounting for 68% of all, while the dinucleotides repeats accounted for 15%, and another polynucleotide SSRs occurred at less frequently (Figure 5D, Tables S7 and S8). From the perspective of species, *C. laevigata* (125) had the most SSRs and *G. cissiformis* var. *cissiformis* (58) had the least (Figure 5C). 

### 2.4. Selective Pressures Events

**Figure 6:**
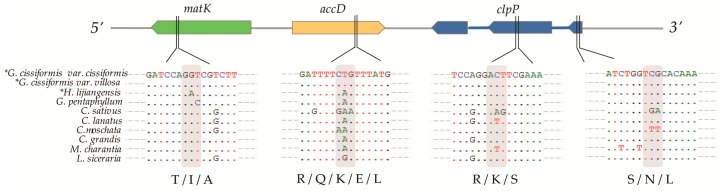
Alignment of selective sites of 10 Cucurbitaceae species. * marked three newly obtained CPGs.

The non-synonymous (*K_A_*) and synonymous (*K_S_*) substitution ratio (*K_A_/K_S_*) were calculated for 68 consensus protein-coding genes to estimate selective pressures. Although all of the *K_A_/K_S_* (ω) values were less than 1.0 in codeml, the *K_A_/K_S_* ratio of five genes (*clpP*, *atpE*, *psbL*, *accD*, and *matK*) were within the range of 0.5 to 1.0 indicating a relaxed selection. Among them, the likelihood ratio test (LRT) analysis showed several sites from three genes (*accD*, *clpP*, and *matK*), which were distributed in the LSC region (Table 3, Figure 6), were under selection. We located the consistent selective sites under the naive empirical Bayes (NEB) and the Bayes empirical Bayes (BEB) methods in the alignment of CPGs, and found these amino acid sites had a high level of variation, for example, in the 308 site of *accD* gene, the codon CGG could have the variables CAG, CTG, AAG, and GAA (Figure 6). Unfortunately, there was only one *K_A_/K_S_* (ω) value that was greater than 1.0 (gene *atpE*), but no significant *p*-value (*p* < 0.05, Table S9) was found using the KaKs-calculator.

### 2.5. Codon Usage Bias and Unconventional Initiation Codon

Codon usage of the protein-coding genes was analyzed in the CPGs of 10 Cucurbitaceae species. The number of encoded codons ranged from 25,922 (*C. sativus*) to 26,828 (*G. pentaphyllum*) (Table S10). Detailed codon analysis showed that the 10 Cucurbitaceae species had a similar codon constituent, and close RSCU (relative synonymous codon usage) values (Table S10). Leucine (Leu) and Cysteine (Cys) were the highest (10.60%) and lowest (1.20%) frequently used amino acids in these species, respectively (Figure S3A, Table S10). The results revealed that most of the amino acid codons have preferences with the exception of Met (Methionine—AUG) and Trp (Tryptophan—UGG). Three newly obtained CPGs had 31 biased codons with RSCU > 1, while other CPGs had 30 (Table S10) due to the difference in codon Ser (Serine—UCC), which were used more than 350 times in the three species and less than 336 times in other species (Table S10). It was illustrated that the genera *Gomphogyne* and *Hemsleya* preferred using Serine more than any other genera. The codons had lower representation rates for C or G at the third codon position, and the average GC content of the third codon base was 37.8%, with the range from 37.6% to 38.0% (Table S11). It turned out that the CPGs of Cucurbitaceae species had a strong bias toward A or T at the third codon position.

**Figure 7:**
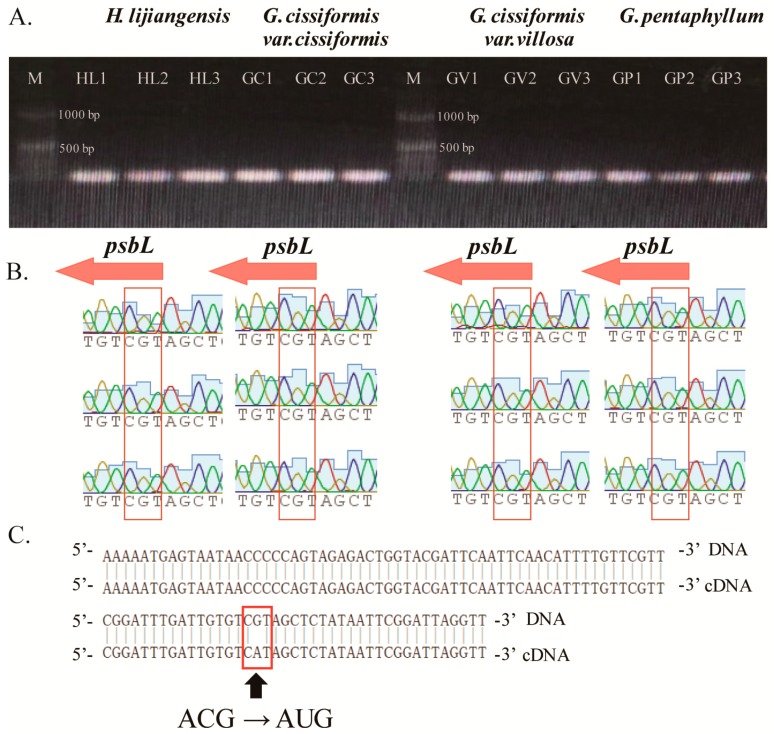
Result of unconventional initiation codon. (**A**) The PCR products electrophoresed in 1% agarose gel; (**B**) The PCR products sequences including start codon of gene *psbL*; (**C**)Blast results of sequence fragment of *G. pentaphyllum* including the start codon of *psbL* in the published transcriptome dataset.

Interestingly, an unconventional initiation codon (Thr-TGC) of *psbL* gene was stumbled on when three new species sequences were annotated. This phenomenon was also found in *G. pentaphyllum* after a global alignment of ten Cucurbitaceae species. When the sequences including the start codon of the *psbL* gene were blasting with the transcriptome dataset of *G. pentaphyllum*, it was indeed found that the ACG start codon had been converted into an initiation codon, AUG (Figure 7). This could be explained by the occurrence of RNA editing phenomenon during the translation process, which reverted this change.

### 2.6. Phylogenetic Analysis

**Figure 8:**
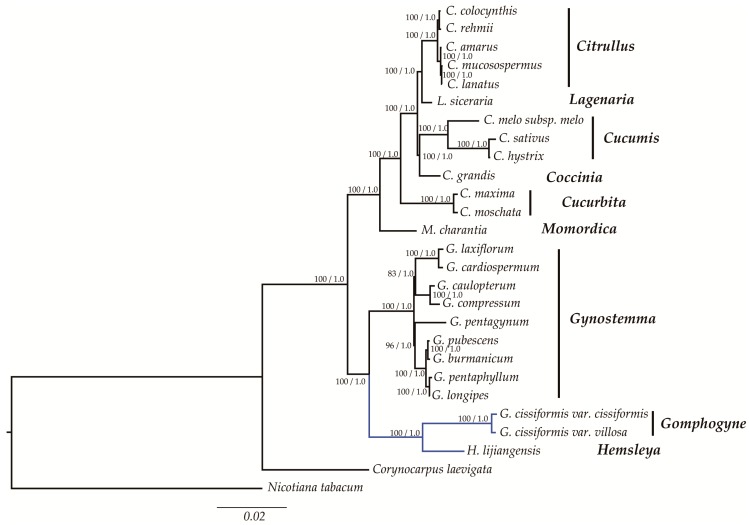
Phylogenetic relationship of the 27 species inferred from ML and BI analyses based on the complete cp genome sequences. The bootstrap values of ML analyses and Bayesian posterior probabilities are shown beside the clades. *Corynocarpus laevigata*, and *Nicotiana abacum* were used as outgroups. The clades in blue color showed the three newly sequenced species in our study.

All the ML and BI trees were reconstructed based on five datasets with the species of Cucurbitaceae released in NCBI. The best-fit models of ML and BI trees using the overall CPGs were GTR + I + G and TVM + I + G, respectively, and that for other datasets were displayed above the tree clade in Figure S4. It was shown that the phylogeny produced from the analyses of 27 complete CPG sequences was well-supported. All nodes of the phylogenetic tree were strongly supported by the 1.00 Bayesian posterior probabilities in BI analysis and 83–100% bootstrap values in ML analysis (Figure 8). It was shown that plants of Cucurbitaceae were clustered into one clade. Genera *Hemsleya* and *Gomphogyne*, constituted the earliest diverging lineage in this group, holding the closest relationship with genus *Gynostemma* and this clade was identified as a sister to all other species. Although there were some variations embodied in the phylogenetic positions of *C. rehmii* in *Citrullus* and *G. pentagynum* in *Gynostemma*, the phylogenetic relationships of any other species were concordant among the genera of Cucurbitales (Figure S4).

## 3. Discussion

### 3.1. Evolution and Variation of Chloroplast Sequences

In angiosperms, most of the CPGs have evolved rapidly [29] and have some structural changes, such as gene rearrangements [30], gene loss-and-gain [31] and gene inversion [32], but no rearrangements events were found in any of our species after global alignment with the published CPGs, even if they contained a large number of large repeat sequences, which may be a reaction to the rearrangement of CPGs and sequence divergence in some other studies [33,34]. All ten CPGs that we studied displayed a typical quadripartite structure, with two SCs and two IRs arranged at regular intervals, and a highly conserved in genome structure and gene order., The pseudogene was initially thought to have lost the ability of protein coding [35] but was, instead, an evolutionary relic of the functional component [36]. In the present study, genes *ycf1* and *rpl2* were both lost in our species, while the *ycf1* gene was found to be a pseudogene in *M. charantia* and *L. siceraria*, but existed in *G. cissiformis* var. *cissiformis*, *G. cissiformis* var. *villosa*, *H. lijiangensis*, and *G. pentaphyllum*. In the complete CPGs of these aforementioned four species, plus *Syzygium cumini* and *Ananas comosus* [37,38], gene *infA* was also regarded as a pseudogene, and it was also found to be lost in the CPGs of *Alstroemeria aurea* and *Arabidopsis thaliana* [39,40].

The IR regions are highly conserved, and they are important in the stabilization of the CPG structure [41]. This is a common evolutionary phenomenon in plants and mainly reflected in the variation of CPGs in length [42,43]. Our results from the comparison of IR/SC boundary areas among species also suggested expansions and contractions of the IR region. As expected, both mVISTA and sequence divergence analysis indicated that CDS and IRs were more conserved than CNS and SCs. The sequence divergence also revealed many significant differences among the CPGs of the family, but a low level of differentiation between species within the genus *Gomphogyne*. When constructing phylogenetic trees with the sequences of four highly variable genes and four highly divergent intergenic regions of each CPG, the results were derived from the phylogenetic analyses based on the entire CPGs (Figure S4). These results indicated that the highly variable regions could be used in the phylogenetic analyses of Cucurbitaceae. Further work is still necessary to determine whether these highly variable regions could serve as candidate DNA barcodes to identify species. SSRs, which are also called microsatellites, can be used to analyze the genetic diversity, population structure, and phylogeography based on polymorphisms [26,44]. Thus, the SSR sequences we identified could contribute to molecular and evolutionary ecological knowledge, which warrants further research at the population level.

### 3.2. Selective Genes and RNA Editing

Analysis of the adaptive evolution of genes has an important reference value in examining the change of gene structure and functional mutations [45]. The percentage of nonsynonymous (*K_A_*) versus synonymous (*K_S_*) nucleotide substitutions (denoted by *K_A_/K_S_*, or ω value) is usually used to evaluate the rate of gene divergence, and determine whether positive, purifying, or neutral selection has been in operation [46]. The *K_A_/K_S_* ratio may reveal the constraints of natural selection on organisms, and the estimation of these mutations contribute greatly to understanding the dynamics of molecular evolution [47]. If ω > 1.0, the corresponding genes experience positive selection, while 0.5 < ω < 1.0, and ω < 0.5 indicate relaxed selection and purifying selection, respectively [48]. Among our calculations, there were five genes under relaxed selection (0.5 < ω < 1.0, Table 3), and several selective sites were found in three (*accD*, *clpP*, and *matK*) of the genes.

It is well established that acetyl-CoA carboxylase (ACCase, EC 6.4.1.2) catalyzes the formation of malonyl-CoA from acetyl-CoA, and it is considered to be the regulatory enzyme of fatty acid synthesis [49,50]. The *accD* gene exactly encodes the β-carboxyl transferase subunit of acetyl-CoA carboxylase [51,52]. It is an essential gene required for leaf development [50], and has great effects on leaf longevity and seed yield [53]. However, this gene has been lost, or defined as a pseudogene, in some species of Primulaceae, Acoraceae, and Poales [22,54]. The *clpP* gene encodes the ATP-dependent clp protease proteolytic subunit [55]. This protein is an essential component to form the protein complex of clp protease (endopeptidase clp) which is active and probably involved in the turnover of chloroplast proteins [56]. It was reported that the loss of *clpP* gene product (the clpP protease subunit) would lead to ablation of the shoot system of tobacco plants, suggesting that clpP-mediated protein degradation is essential for shoot development [57,58]. The *matK* (maturase K) gene is a plant chloroplast gene [59] which is located within the intron of the *trnK* gene (Figure 1). The protein it encodes is an intron maturase which is involved in the cutting and splicing of Group II RNA transcriptional introns [60,61]. The *matK* retains only a well-conserved domain X, and remnants of a reverse transcriptase domain [61]. Usually, the *matK* gene sequence is effectively used as a DNA barcoding fragment for angiosperms, in studies of plant systematics [62,63,64].

In summary, the coding proteins of these selective genes were all enzymes functioning in chloroplast protein synthesis, gene transcription, energy transformation, and plant development. The majority of wild species in Cucurbitaceae are creeping herbs, mainly distributed in moist mountains, forests, thickets, and streamside, and they may have some mechanisms for adapting to complex living conditions. Therefore, the species may have produced some corresponding differentiation in morphology during the long process of evolution. Consequently, we inferred that the chloroplast functional genes which were under selection might play key roles during the adaptation and development of the Cucurbitaceae species to terrestrial ecosystems.

Codon usage bias plays an important role in the evolution of CPG. The main factor that contributed to biased codon usage is the GC content, which is also important during the evolution of genomic structure, such as stability of replication, transcription, and translation [65,66]. The observed GC content level indicated that the CPGs in Cucurbitaceae were GC-lacking, and that there was a strong bias towards A/T at the third codon position, consistent with the existing CPG research [67,68,69,70]. The presence of translation-preferred codons might be the result of both mutation preference and natural selection during the CPG evolutionary process [71].

The genetic information in land plant chloroplast DNA is sometimes altered at the transcript level by a process known as RNA editing [72]. This process of the post-transcriptional modification of precursor RNAs to alter their nucleotide sequences [73]. It sometimes occurs through the insertion and deletion of nucleotides, or specific nucleotide substitution (mostly C to U conversion) [72]. Since the first evidence of RNA editing was found in chloroplast in the rpl2 transcript of maize [74], it has been hunted out and systematically studied in the protein-coding transcripts from many major lineages of land plants [75], such as *Arabidopsis thaliana* [76], *N. tabacum* [49], *Zea mays* [77], *Oryza sativa* [78], *Cucumis melo*, and *Cucurbita maxima* [79]. Most of the studies suggested that RNA editing occasionally created start or stop codons which shorten the size of translation products [72,80,81], even producing a new gene in one striking case [80]. Our results revealed an unconventional initiation codon in the *psbL* gene, having a function in producing the PSII-L protein in the photosystem II (PSII) complex [82], caused by nucleotide substitution which was generated by RNA editing on the second position of start codon (ACG to AUG). This phenomenon was also found in bell pepper [83], tobacco [84,85], spinach [73], and *Ampelopsis brevipedunculata* [86]. RNA editing is very common in plant chloroplast genomes. It can modify mutations, change reading frames, and regulate the expression of chloroplast genes [87], acting as a correction mechanism in the chloroplast of plants.

### 3.3. Phylogeny of Cucurbitaceae

The number of studies using complete CPG sequences for assessing phylogenetic relationships among angiosperms has been increasing rapidly [21,88,89,90]. Our phylogenetic trees (both ML and BI trees) indicated a clear relationship of the genera in Cucurbitaceae with high bootstrap values. The phylogenetic trees demonstrated that the genera *Gomphogyne*, *Hemsleya*, and *Gynostemma* constituted the earliest diverging lineage in Cucurbitaceae. This was consistent with the proposal that these three genera were relatively original genera belonging to the family Cucurbitaceae based on morphology [91]. All in all, our results suggested that the CPG data can effectively resolve the phylogenetic relationships of these genera in Cucurbitaceae. In fact, for this large family, our study was just a drop in the bucket. Some studies pointed out that the lack of samples might also affect the results of the phylogenetic analysis [22]. Unfortunately, our study could not roundly figure out the relationships among genera due to the limited sample size. More species from more genera should be included in the future. Furthermore, our phylogenetic study was based solely on chloroplast DNA. In order to comprehensively understand of the systematic evolution of Cucurbitaceae, nuclear DNA analyses are required to investigate the effect of gene introgression and hybridization on phylogeny. Our phylogenetic studies provided a valuable resource that should contribute to the future taxonomy, phylogeny, and evolutionary history studies of the Cucurbitaceae family.

## 4. Materials and Methods

### 4.1. Plant Materials and DNA Extraction

Healthy leaves of three species (*G. cissiformis* var. *cissiformis*, *G. cissiformis* var. *villosa*, and *H. lijiangensis*) were collected from adult plants in Yunnan province, China (Table 1). Voucher specimens were deposited in the Evolutionary Botany Laboratory of Northwest University (Shaanxi, China). Total genomic DNA were extracted from silica-dried leaf materials with simplified CTAB protocol [92]. Data from seven complete CPGs (Table 1) [26,93,94,95] were recovered from the National Center of Biotechnology Information (NCBI) in order to conduct the follow-up analyses.

### 4.2. Illumina Sequencing, Assembly, and Annotation

Illumina raw reads were collected using an Illumina Hiseq 2500 platform. The quality-trim with all of the raw reads was performed using CLC Genomics Workbench v7.5 (CLC bio, Aarhus, Denmark) with the default parameter set. The programs MITObim v1.7 (University of Oslo, Oslo, Norway) [96] and MIRA v4.0.2 (DKFZ, Heidelberg, Germany) [97] were used to perform the reference-guided assembly twice, to reconstruct the CPGs with published *G. pentaphyllum* (KX852298) and *C. melo* (JF412791) as references, respectively. A few gaps, dubious bases, and low-coverage regions in the assembled CPGs were corrected by Sanger sequencing, whereby pairs of primers were designed (Table S1) using Primer 3 version 4.0.0 (Whitehead Institute for Biomedical Research, Massachusetts, USA) [98]. The software DOGMA, Dual Organellar Genome Annotator (University of Texas at Austin, Austin, TX, USA) [99], was used to annotate the complete CPGs, and corrected by comparing with the complete CPGs of the references mentioned above using GENEIOUS R8 (Biomatters Ltd., Auckland, New Zealand). The circular CPG maps were drawn using online software OGDRAW (http://ogdraw.mpimp-golm.mpg.de) (Max planck Institute of Molecular Plant Physiology, Potsdam, Germany). All of the newly generated complete CPG sequences were submitted to GenBank (Table 1).

### 4.3. Comparison of Complete Chloroplast Genomes

The mVISTA (The Regents of the University of California, Oakland, CA, USA) [100] software was employed to discover the interspecific variation among the complete CPG sequences of eleven species (ten Cucurbitaceae species and *Corynocarpus laevigata*, Corynocarpaceae, HQ207704), and the alignments with annotations were visualized using *G. cissiformis* var. *cissiformis* as reference. In order to analyze the expansions and contractions, as well as the variation in junction regions among ten Cucurbitaceae species, the IR region borders and gene rearrangements were surveyed by the plug-in program, Mauve, in GENEIOUS R8. To analyze the bivariate correlational relationship between the overall CPG sizes and each of the structural regions of CPGs, i.e., LSC region, SSC region and IR region, we used IBM SPSS Statistics v21.0 (SPSS Inc., Chicago, IL, USA) with Pearson’s one-tail test, and the significant value was *p* < 0.05.

### 4.4. Sequence Divergence

The multiple alignments of the CPGs were carried out using MAFFT version 7.017 (Osaka University, Suita, Japan) [101]. DnaSP v5.0 (Universitat de Barcelona, Barcelona, Spain) [102] was used to compute the variable sites across the complete CPGs, LSC, SSC, and IR regions of all the species. To investigate the sequence divergence patterns, MEGA 5.0 (Tokyo Metropolitan University, Tokyo, Japan) [103] was employed for statistical analysis of the variations of CPGs and percentage of indels among each region. The percentage of variable characters for each coding and noncoding region were calculated based on the method of Zhang [104]. 

### 4.5. Microsatellites and Repeated Sequences 

Microsatellites (SSRs) and three categories of repeated sequences were detected in all eleven Cucurbitaceous species. The software, MISA (Institute of Plant Genetics and Crop Plant Research, Gatersleben, Germany)) [105] was utilized to seek the microsatellites (SSRs) with thresholds of 10, 5, 4, 3, 3, and 3, for mono-, di-, tri-, tetra-, penta-, and hexa-nucleotides, respectively. The online program, Tandem Repeats Finder (http://tandem.bu.edu/trf/trf.html) (Mount Sinai School of Medicine, New York, NY, USA) [106], was used to find the tandem repeat sequences, which were at least 10 bp in length. The alignment parameters for match, mismatch, and indels were set to be 2, 7, and 7, respectively. To search out the size and location of dispersed and palindromic repeats, the online program REPuter (https://bibiserv2.cebitec.uni-bielefeld.de/reputer) (University of Bielefeld, Bielefeld, Germany) [107] was performed with parameters of 30 bp minimal repeat size, and the similarity percentage of two repeat copies was set to at least 90%. 

### 4.6. Selective Pressure Analysis

Selective pressures were analyzed for consensus protein-coding genes among ten Cucurbitaceae species. PAML with codeml program (University College London, London, UK) [108] was performed to calculate the nonsynonymous (K_A_) and synonymous (K_S_) substitution ratio. In order to estimate the ω value (ω = K_A_/K_S_) of every gene sequence, the method reported by Yang and Nielsen [109] was adopted. Adaptive evolution of genes was confirmed by computing likelihood ratio tests (LRTs). The KaKs-calculator (Chinese Academy of Sciences, Beijing, China) [110] was also used to calculate K_A_, K_S_, and the K_A_/K_S_ ratio, based on a model-averaging method.

### 4.7. Codon Usage Bias and Unconventional Initiation Codon

Codon usage and RSCU values [111] were estimated for all exons in the consensus protein-coding genes with the CodonW v1.4.2 program (University of Nottingham, Nottingham, UK) [112]. For the purpose of verifying the existence of unconventional initiation codon, we designed pairs of primers (Table S1) for polymerase chain reaction (PCR) amplification of target fragments within four species (three new species and *G. pentaphyllum*). The products of PCR were tested using 1% agarose gel electrophoresis and sequenced. The obtained sequences were mapped to the corresponding CPGs using the software GENEIOUS R8 (Biomatters Ltd., Auckland, New Zealand). Due to the lack of RNA sequence data, we blasted a 101 bp CPG sequence fragment of *G. pentaphyllum*, including the start codon of *psbL* gene, in the published transcriptome dataset of *G. pentaphyllum* (accession number in NCBI: SRX1364750) [113] using the online program BLASTn (https://blast.ncbi.nlm.nih.gov; U.S. National Library of Medicine, Bethesda, Rockville, MD, USA).

### 4.8. Phylogenetic Relationships

To reconstruct the phylogenetic relationship, 15 published complete CPG sequences from Cucurbitales were also selected in the analyses (Table S2). In total, 27 sequences were aligned using the MAFFT v7.017 program (Osaka University, Suita, Japan) [101]. Due to the differentiation of the molecular evolutionary rate among the different CPG regions, phylogenetic relationship analyses were performed using the following five datasets: (1) the overall CPG sequences; (2) the large-single copy region (LSC); (3) the small single-copy region (SSC); (4) one inverted repeats region (IRb); (5) consensus protein coding genes (CDS); and (6) eight highly variable regions (HVR). The best-fitting model for each dataset was determined by software Modeltest 3.7 (Brigham Young University, Provo, UT, USA) [114] under the Akaike information criterion. Bayesian inference (BI) was performed by MrBayes 3.12 (SwedishMuseum of Natural History, Stockholm, Sweden) [115] using the following parameters: Markov chain Monte Carlo simulations algorithm (MCMC) for 1 × 10^5^ generations with four incrementally-heated chains. The maximum likelihood (ML) trees were implemented with RAxML v7.2.8 (Heidelberg Institute for Theoretical Studies, Heidelberg, Germany) [116] with 1000 replicates. In all analyses, *C. laevigata* and *N. tabacum* (Z00044) were chosen as outgroups.

## 5. Conclusions

The comparative analyses of complete CPGs contribute towards understanding the complete CPG structure and evolution, the identification of species, and the determination of phylogenetic relationships. Here, we have successfully applied Illumina sequencing to determine the complete CPGs of three herbaceous plants from the Cucurbitaceae, further enriching the valuable resources for the complete CPGs of higher plants. The results revealed that they shared most of the common genomic features with other species of Cucurbitaceae. Sequence divergence analysis showed high conservatism of the coding and IR regions. The coding proteins of three selective genes (*accD*, *clpP* and *matK*) were screened out, and they would contribute to analyzing the adaptive evolution. Evidence for RNA editing was demonstrated involving an unconventional initiation codon in the *psbL* gene. Phylogenetic analyses revealed that the genera *Gomphogyne*, *Hemsleya*, and *Gynostemma* were the earliest diverging lineage in Cucurbitaceae. The study suggested that the complete chloroplast genome sequences were useful for phylogenetic studies. This would enrich the valuable complete chloroplast genome resources of Cucurbitaceae, and determine potential SSR molecular markers and candidate DNA barcodes for coming phylogenetic and evolutionary population studies.

## Figures and Tables

**Table 1:** Genome features of the chloroplast genomes of ten Cucurbitaceae species.

**Species**	* *G. cissiformis* var. *cissiformis*	* *G. cissiformis* var. *villosa*	* *H. lijiangensis*	***G. pentaphyllum***	***C. lanatus***
Locations	24.20° N, 99.50° E	24.20° N, 99.50° E	27.17° N, 100.06° E	/	/
Assembly reads	1,274,004	1,505,442	1,483,091	/	/
Mean coverage	1112.4	1358.4	1392.7	/	/
Size (bp)	157,334	156,585	158,275	157,576	156,906
LSC (bp)	87,239	86,642	87,362	86,757	86,845
SSC (bp)	18,014	18,029	18,429	18,653	17,897
IRs (bp)	26,041	25,957	26,242	26,083	26,082
Number of total genes	133	133	133	133	122
Number of protein-coding genes	87	87	87	87	85
Number of tRNA genes	37	37	37	37	29
Number of rRNA genes	8	8	8	8	8
Pseudogene	*infA*	*infA*	*infA*	*infA*	/
Overall GC content (%)	37	37	37	37	37.2
GC content in LSC (%)	34.8	34.8	34.8	34.8	34.9
GC content in SSC (%)	31.1	31	31	30.6	31.5
GC content in IR (%)	42.8	42.7	42.8	42.8	42.8
GenBank number	MH256801	MF784515	MG733988	KX852298	KY014105
**Species**	***C. grandis***	***C. sativus***	***C. moschata***	***M. charantia***	***L. siceraria***
Locations	/	/	/	/	/
Assembly reads	/	/	/	/	/
Mean coverage	/	/	/	/	/
Size (bp)	157,035	155,293	157,644	158,844	157,145
LSC (bp)	86,749	86,688	88,343	88,374	86,843
SSC (bp)	18,004	18,223	18,156	18,010	18,008
IRs (bp)	26,141	25,193	25,573	26,228	26,147
Number of total genes	132	132	135	130	130
Number of protein-coding genes	85	85	85	85	86
Number of tRNA genes	39	38	42	38	37
Number of rRNA genes	8	8	8	8	8
Pseudogene	/	*rps16*	/	*ycf1*	*ycf1*
Overall GC content (%)	37.1	37.1	37.1	36.7	37.1
GC content in LSC (%)	34.8	34.8	34.9	34.3	34.9
GC content in SSC (%)	31.3	31.8	31.5	30.7	31.4
GC content in IR (%)	42.8	42.8	43.1	42.8	42.8
GenBank number	KX147312	AJ970307	MF991116	MG022622	MG022623

* Three newly obtained chloroplast genomes.

**Table 2:** List of genes in the chloroplast genome of three newlysequenced species.

Category	Gene Group	Gene Name
Self-replication	Ribosomal protein (small subunit) (14)	rps2 rps3 rps4 rps7 (×2) rps8 rps11 * rps12 (×2) rps14 rps15 * rps16 rps18 rps19
	Ribosomal protein (large subunit) (11)	* rpl2 (×2) rpl14 * rpl16 rpl20 rpl22 rpl23 (×2) rpl32 rpl33 rpl36
	RNA polymerase (4)	rpoA rpoB * rpoC1 rpoC2
	Transfer RNAs (37)	* trnA-UGC (×2) trnC-GCA trnD-GUC trnE-UUC trnF-GAA trnfM-CAU * trnG-UCC trnG-GCC trnH-GUG trnI-CAU(×2) * trnI-GAU (×2) * trnK-UUU trnL-CAA(×2) trnL-UAG * trnL-UAA trnM-CAU trnN-GUU(×2) trnP-UGG trnQ-UUG trnR-ACG(×2) trnR-UCU trnS-GCU trnS-GGA trnS-UGA trnT-GGU trnT-UGU trnV-GAC(×2) * trnV-UAC trnW-CCA trnY-GUA
	Ribosomal RNAs (8)	*rrn4.5(×2) rrn5(×2) rrn16(×2) rrn23(×2)*
Photosynthesis	Photosystem I (5)	*psaA psaB psaC psaI psaJ*
	Photosystem II (15)	*psbA psbB psbC psbD psbE psbF psbH psbI psbJ psbK psbL psbM psbN psbT psbZ*
	Cytochrome b/f complex (6)	petA * petB * petD petG petL petN
	ATP synthase (6)	atpA atpB atpE * atpF atpH atpI
	NADH dehydrogenase (12)	* ndhA * ndhB (×2) ndhC ndhD ndhE ndhF ndhG ndhH ndhI ndhJ ndhK
	Rubisco large subunit (1)	*rbcL*
Other genes	Maturase (1)	*matK*
	membrane protein (1)	*cemA*
	Acetyl-CoA carboxylase gene (1)	*accD*
	ATP-dependent protease subunit (1)	*clpP*
	c-type Cytochrome biogenesis (1)	*ccsA*
	Assembly/stability of photosystem I (2)	*ycf3 ycf4*
	Conserved reading frames (ycfs) (4)	*ycf1(×2) ycf2(×2)*
	hypothetical chloroplast protein (2)	*orf70(×2)*
Pseudogene	Translation-related gene (1)	*infA*

* Gene with intron(s).

**Table 3:** Parameter estimates and log-likelihood values for different models in selective pressure analysis.

Genes	Model	df	lnL/ω Value	LRTs	No. of Sites (BEB)	Consistent Sites
***clpP***	M0 (one ratio)	19	**ω = 0.96975**			
	M1 (neutral)	20	−1396.7593	M1 vs. M2:	2	12 S/N/L
	M2 (selection)	22	−1389.6667	14.1852 **		AGT/AAT/CTT
	M7 (beta)	20	−1396.7638	M7 vs. M8:	4	87 R/K/S
	M8 (beta&ω)	22	−1389.6672	14.1933 **		CGA/AAA/TCA
***atpE***	M0 (one ratio)	19	**ω = 0.70941**			
	M1 (neutral)	20	−787.914712	M1 vs. M2:	0	/
	M2 (selection)	22	−785.703588	4.42225		
	M7 (beta)	20	−787.942937	M7 vs. M8:	0	
	M8 (beta&ω)	22	−785.704169	4.47754		
***psbL***	M0 (one ratio)	19	**ω = 0.61775**			
	M1 (neutral)	20	−158.807141	M1 vs. M2:	0	/
	M2 (selection)	22	−158.807091	0.00010		
	M7 (beta)	20	−158.807137	M7 vs. M8:	0	
	M8 (beta&ω)	22	−158.80712	0.00003		
***accD***	M0 (one ratio)	19	**ω = 0.53161**			
	M1 (neutral)	20	−3221.0459	M1 vs. M2:	1	308 R/Q/K/E/L
	M2 (selection)	22	−3214.0011	14.0896 **		CGG/CAG/CTG/AAG/GAA
	M7 (beta)	20	−3221.5205	M7 vs. M8:	4	
	M8 (beta&ω)	22	−3214.0768	14.8875 **		
***matK***	M0 (one ratio)	19	**ω = 0.52255**			
	M1 (neutral)	20	−3688.5152	M1 vs. M2:	1	337 T/I/A
	M2 (selection)	22	−3683.8284	9.3735 **		TCA/GGA/GCA
	M7 (beta)	20	−3689.0422	M7 vs. M8:	8	
	M8 (beta&ω)	22	−3683.9212	10.2421 **		

** *p* < 0.01; df: degree of freedom; the likelihood ratio tests, LRT = |df(M2/M8) − df(M1/M7)| × |lnL(M2/M8) − lnL(M1/M7)|; No. of Sites: the number of selective sites under the Bayes empirical Bayes (BEB) model; Consistent sites: the sites appeared in both M1 vs. M2 and M7 vs. M8, showing the amino acids and their corresponding codons.

## Supplementary Materials

The following are available online. Figure S1. Genome rearrangement events of 10 Cucurbitaceae species, comparing with *C. laevigata* and *N. tabacum*; Figure S2. Sequence identity plots among 11 chloroplast genomes, with *G. cissiformis* var. *cissiformis* as a reference by using mVISTA. The vertical scale indicates the identity percentage ranging from 50% to 100%. The horizontal axis corresponds to the coordinates within the chloroplast genome. Coding and non-coding regions are marked in blue and pink, respectively. Annotated genes are displayed along the top. The black boxes show the two IR regions; Figure S3. Amino acid frequencies and base compositions of chloroplast genomes of four species. (A) Frequency of amino acids usage; (B) Base compositions of chloroplast genomes; Figure S4. Phylogenetic relationship of the 27 species inferred from ML and BI analyses based on four datasets (A. LSC; B. SSC; C. IR; D. CDS; E. 8 HVR). The bootstrap values of ML analyses and Bayesian posterior probabilities are shown beside the clades. *C. laevigata* and *N. tabacum* were used as the outgroups; Table S1. Primers for low coverage regions of Cucurbitaceae species; Table S2. List of species and their basic information included in the analyses of complete chloroplast genomes; Table S3. Percentages of variable characters in coding and non-coding regions and the percentage of indels in variable characters; Table S4. PCR primers for relatively highly variable regions; Table S5. List of tandem repeats in the chloroplast genome of eleven Cucurbitales species; Table S6. List of dispersed repeats and palindromic repeats in the chloroplast genome of eleven Cucurbitales species; Table S7. List of SSRs in the chloroplast genome of eleven Cucurbitales species; Table S8. Summary of three categories of repeats and simple sequence repeats (SSRs); Table S9. Results of selective events analysis in KaKs-calculator; Table S10. Codon usage and relative synonymous codon usage (RSCU) value for protein-coding genes in the ten Cucurbitaceae chloroplast genomes; Table S11. Base compositions for protein-coding genes in the ten Cucurbitaceae chloroplast genomes.
