# Supplementary material for: Comparative Analyses of Chloroplast Genomes of Cucurbitaceae Species: Lights into Selective Pressures and Phylogenetic Relationships
Source: Molecules. 2018 Aug 28;23(9):2165. doi: 10.3390/molecules23092165 (PMC6225112; doi:10.3390/molecules23092165)
Supplement: Supplementary file 1 [file molecules-23-02165-s001.pdf]

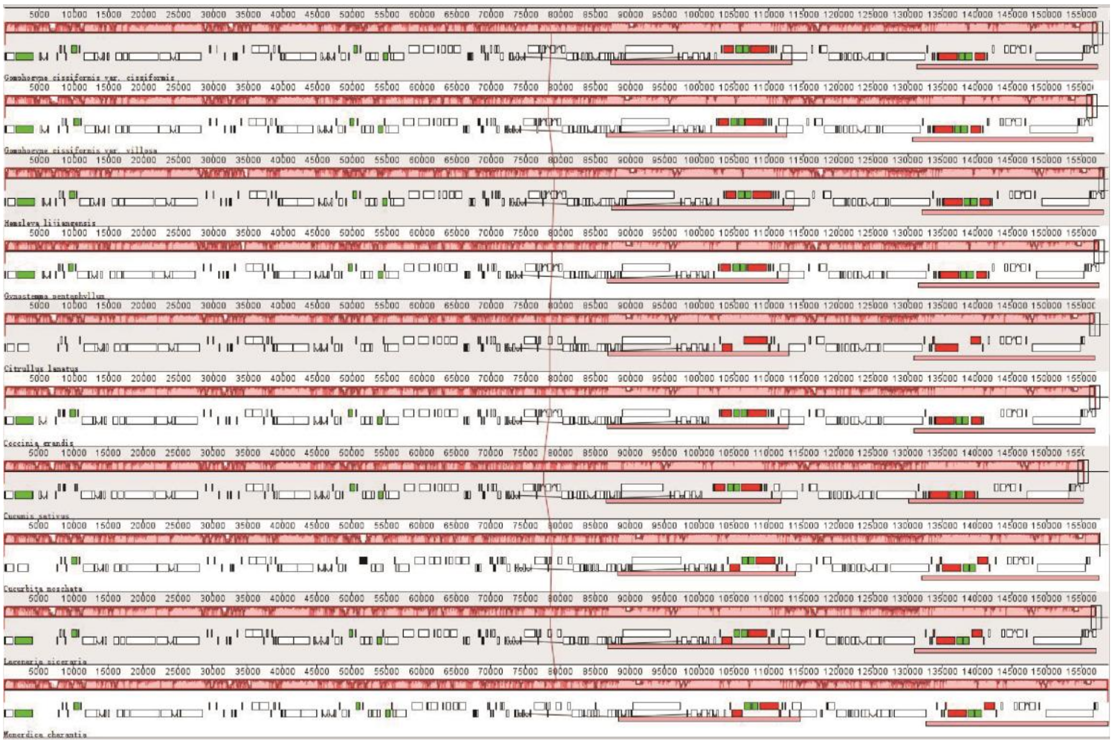

Figure S1. Genome rearrangement events of 10 Cucurbitaceae species, comparing with *C. laevigata* and *N. tabacum.*

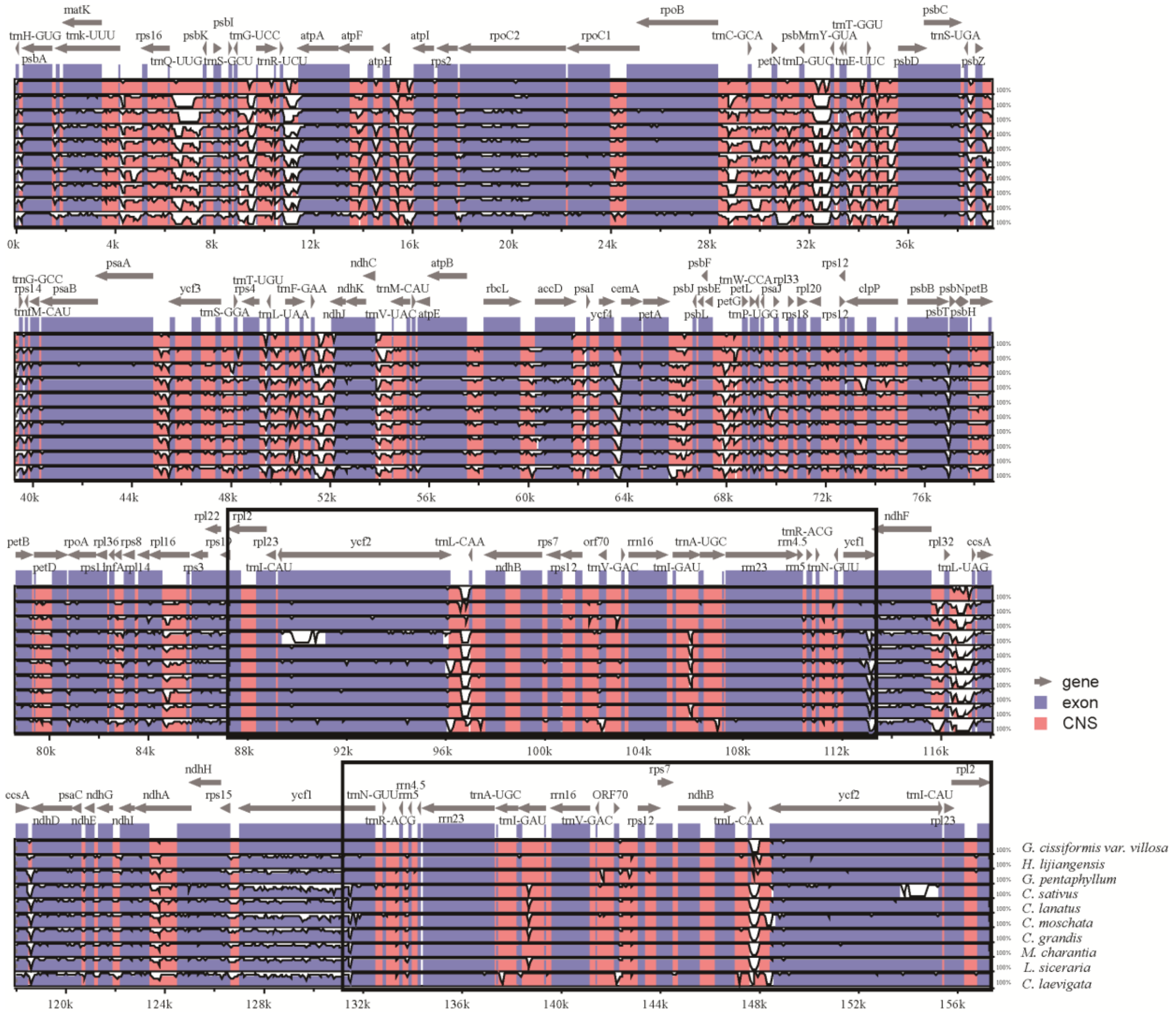

Figure S2. Sequence identity plots among 11 chloroplast genomes, with *G. cissiformis var. cissiformis* as a reference by using mVISTA. The vertical scale indicates the identity percentage ranging from 50% to 100%. The horizontal axis corresponds to the coordinates within the chloroplast genome. Coding and non-coding regions are marked in blue and pink, respectively. Annotated genes are displayed along the top. The black boxes show the two IR regions.

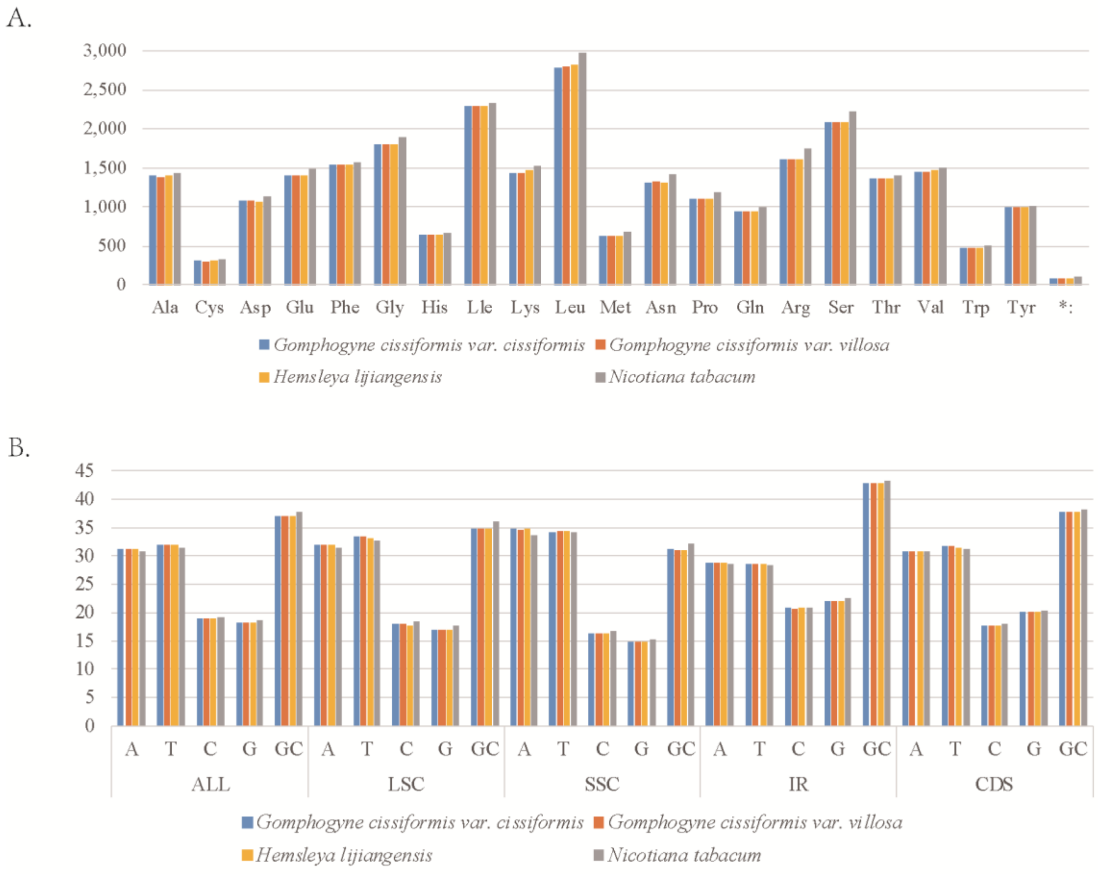

Figure S3 Amino acid frequencies and base compositions of chloroplast genomes of four species. (A) Frequency of amino acids usage; (B) Base compositions of chloroplast genomes.

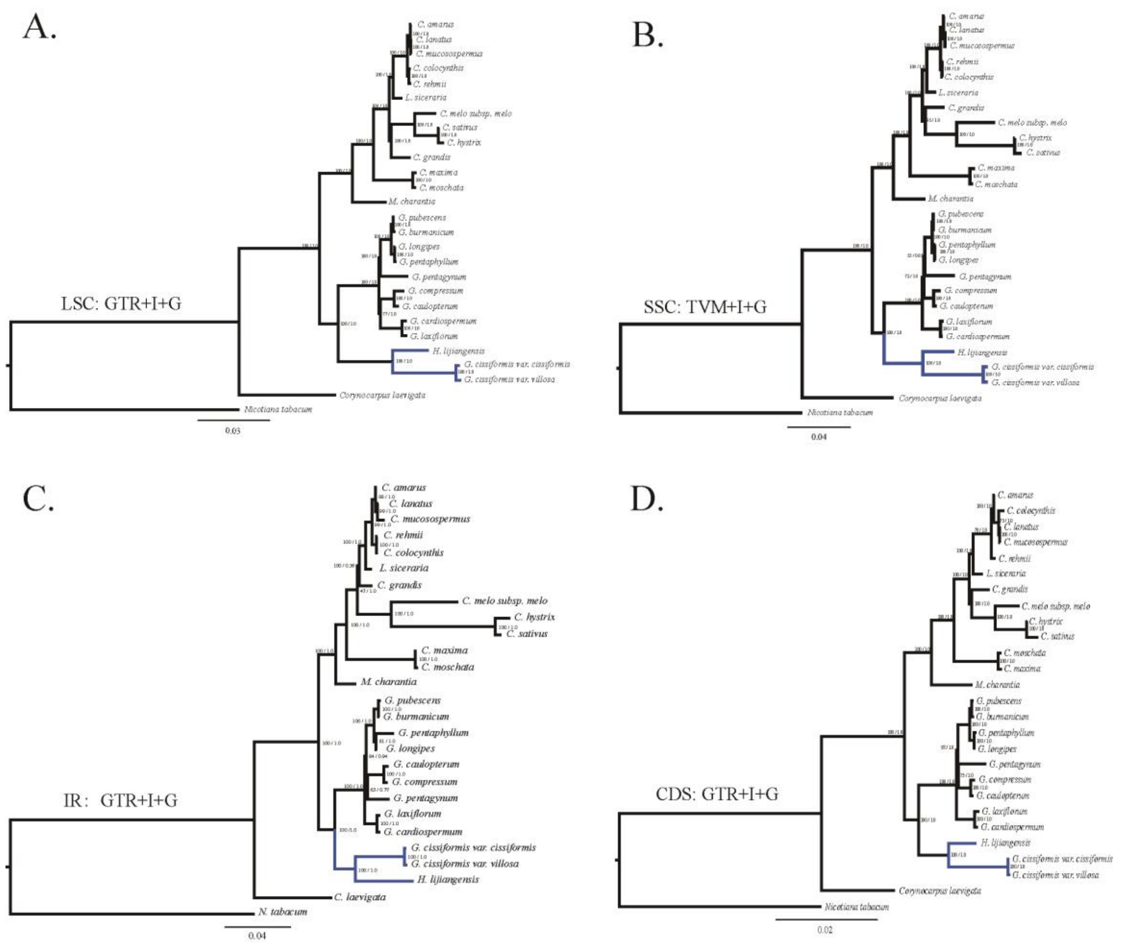

Figure S4 Phylogenetic relationship of the 27 species inferred from ML and BI analyses based on four datasets (A. LSC; B. SSC; C. IR; and D. CDS). The bootstrap values of ML analyses and Bayesian posterior probabilities are shown beside the clades. *C. laevigata* and *N. tabacum* were used as the outgroups.

Table S1. Primers for low coverage regions of Cucurbitaceae species.

No.	Region amplified	Forward sequence (5' to 3')	Reverse sequence (5' to 3')	*Ta* (℃)
G.cissiformis var. cissiformis				
1	*trnfM-rps14*	ACAGAATGGTAAAGGGGTCCC	CCGGACACAAACTTCGTGAA	57
2	*rpl2*	TGTCCACCCTTTCCAAGTGT	CAAAGAGAGGAGGACGGGTT	54
3	*ycf2*	TTTTGGACCTTAGCGCCATG	ATGCCCGTTGAATTGGAAGG	58
4	*trnI-GAU*	TATTCACTCGTGGGATCCGG	CCTCGCCCGTGAAGTAAATC	56
G.cissiformis var. villosa				
1	*ycf2*	TCAGCCTTTTGTCTCTGTGTT	CACCCGTAACCCAGCAGATA	57
2	*trnI-GAU*	TCTCGAGCACAGGTTTAGGT	TCCTTTTCGGGAGCGATTCA	58
3	*trnF-GAA*	TCCTCGTGTCACCAGTTCAA	GGGTATATCTCCAGACATCACGA	55
H.lijiangensis				
1	*rps12-orf70*	TGACGGGTTAGTGTGAGCTT	ATACTTTCCCCGGTTCCGTT	54
2	*atpF*	CATTTGGCTCTCACGCTCAA	AGGTACATGATCCCGCGAAT	55
3	*trnL-UAA-trnF-GAA*	CGATTCCAATATCTCCGAGTTGA	CCACGAAATTTAAGGGCTTGGA	51
4	*psaA-ycf3*	GACTGATGAGGACAACAACCA	TCAAGCCGCTGAGTATTGGA	57
**Universal**				
1	*ndhF*	ACTTCTTACCCTCCGGATGG	TGGTCATACAATCGTGGTTACA	54
2	*psbL*	AGAGACTGGTACGATTCAATTCA	GGGTCAATATCAGCAATGCAGT	54
3	*orf70-trnV*	TGAATCATCCTTTCCACGGAG	TGATGACTTCCACCACGTCA	56
4	*rrn4.5-rrn5*	GGGATGGAGCGACAGAAGTT	AGTTCGGGATGGATTGGTGT	60
5	*trnP-psaJ*	AGGTAGGGATGACAGGATTTGA	TACTTACTACCGGTGCCACG	53

Table S2. List of species and their basic information included in the analyses of complete chloroplast genomes.

No.	Species	genus	Family	Length	Gene	PCG	Accession number
**1**	*G. cissiformis var. cissiformis*	*Gomphogyne*	Cucurbitaceae	157,334	133	87	MH256801
**2**	*G. cissiformis var. villosa*	*Gomphogyne*	Cucurbitaceae	156,585	133	87	MF784515
**3**	*H. lijiangensis*	*Hemsleya*	Cucurbitaceae	158,275	133	87	MG733988
**4**	*G. pentaphyllum*	*Gynostemma*	Cucurbitaceae	157,576	133	87	KX852298
**5**	*G. compressum*	*Gynostemma*	Cucurbitaceae	157,959	133	87	KY817143
**6**	*G. longipes*	*Gynostemma*	Cucurbitaceae	157,601	133	87	MF152730
**7**	*G. pubescens*	*Gynostemma*	Cucurbitaceae	157,666	133	87	MF152732
**8**	*G. burmanicum*	*Gynostemma*	Cucurbitaceae	157,687	133	87	MF152731
**9**	*G. cardiospermum*	*Gynostemma*	Cucurbitaceae	158,219	133	87	KX852299
**10**	*G. laxiflorum*	*Gynostemma*	Cucurbitaceae	158,273	133	87	MF136486
**11**	*G. caulopterum*	*Gynostemma*	Cucurbitaceae	157,937	133	87	MF136487
**12**	*G. pentagynum*	*Gynostemma*	Cucurbitaceae	157,697	133	87	KY670737
**13**	*C. grandis*	*Coccinia*	Cucurbitaceae	157,035	132	75	KX147312
**14**	*C. sativus*	*Cucumis*	Cucurbitaceae	155,293	132	85	AJ970307
**15**	*C. hystrix*	*Cucumis*	Cucurbitaceae	155,031	132	79	NC_023544
**16**	*C. melo subsp. melo*	*Cucumis*	Cucurbitaceae	156,017	132	88	JF412791
**17**	*C. lanatus*	*Citrullus*	Cucurbitaceae	156,906	122	85	KY014105
**18**	*C. mucosospermus*	*Citrullus*	Cucurbitaceae	156,905	122	75	KY430687

**19**	*C. colocynthis*	*Citrullus*	Cucurbitaceae	157,147	131	86	MF357889
**20**	*C. amarus*	*Citrullus*	Cucurbitaceae	157,008	130	85	MF536694
**21**	*C. rehmii*	*Citrullus*	Cucurbitaceae	157,135	130	85	MF536695
**22**	*C. maxima*	*Cucurbita*	Cucurbitaceae	157,204	130	85	MF991115
**23**	*C. moschata*	*Cucurbita*	Cucurbitaceae	157,644	135	85	MF991116
**24**	*M. charantia*	*Momordica*	Cucurbitaceae	158,844	130	85	MG022622
**25**	*L. siceraria*	*Lagenaria*	Cucurbitaceae	157,145	130	86	MG022623
**26**	*C. laevigata*	*Corynocarpus*	Corynocarpus	159,202	128	83	HQ207704
**27**	*N. tabacum*	*Nicotiana*	Solanaceae	155,943	143	111	Z00044

PCG: Number of protein-coding genes.

Table S3. Percentages of variable characters in coding and non-coding regions and the percentage of indels in variable characters.

	CDS	Percentage of variable characters between two Gomphogyne species	Percentage of indels in each variation between two Gomphogyne species	Percentage of variable characters among ten Cucurbitaceae species	Percentage of indels in each variation among ten Cucurbitaceae species		CNS	Percentage of variable characters between two Gomphogyne species	Percentage of indels in each variation between two Gomphogyne species	Percentage of variable characters among ten Cucurbitaceae species	Percentage of indels in each variation among ten Cucurbitaceae species
LSC	*trnH-GUG*	0.000	0.000	0.013	0.000	LSC	*rpl2-trnH (GUG)*	0.000	0.000	0.647	0.025
	psbA	0.000	0.000	0.045	0.063		*trnH (GUG)-psbA*	0.000	0.000	0.736	0.883
	trnk-UUU	0.000	0.000	0.014	0.000		*psbA-trnK (UUU)*	0.004	0.000	0.411	0.305
	matK	0.000	0.000	0.150	0.018		*trnK (UUU)-matK*	0.000	0.000	0.272	0.184
	rps16	0.000	0.000	0.082	0.000		*matK-trnK (UUU)*	0.006	0.000	0.197	0.243
	trnQ-UUG	0.000	0.000	0.000	0.000		*trnK (UUU)-rps16*	0.092	0.989	0.718	0.467
	psbK	0.000	0.000	0.081	0.000		*rps16 intron*	0.002	0.000	0.267	0.431
	psbI	0.000	0.000	0.072	0.000		*rps16-trnQ (UUG)*	0.106	0.988	0.828	0.423
	trnS-GCU	0.000	0.000	0.034	0.000		*trnQ (UUG)-psbK*	0.005	0.000	0.238	0.327
	trnG-UCC	0.000	0.000	0.028	0.000		*psbK-psbI*	0.000	0.000	0.320	0.246
	trnR-UCU	0.000	0.000	0.014	0.000		*psbI-trnS (GCU)*	0.018	1.000	0.345	0.205

atpA	0.000	0.000	0.082	0.000
atpF	0.002	0.000	0.084	0.128
atpH	0.000	0.000	0.049	0.000
atpI	0.000	0.000	0.054	0.000
rps2	0.000	0.000	0.058	0.000
rpoC2	0.002	0.750	0.100	0.010
rpoC1	0.000	0.000	0.057	0.000
rpoB	0.000	0.000	0.055	0.017
trnC-GCA	0.000	0.000	0.042	0.000
petN	0.000	0.000	0.044	0.000
psbM	0.000	0.000	0.057	0.000
trnD-GUC	0.000	0.000	0.014	0.000
trnY-GUA	0.000	0.000	0.000	0.000
trnE-UUC	0.000	0.000	0.000	0.000
trnT-GGU	0.000	0.000	0.000	0.000
psbD	0.000	0.000	0.043	0.000
psbC	0.001	0.000	0.045	0.016
trnS-UGA	0.000	0.000	0.000	0.000

trnS (GCU)-trnG (UCC)	0.085	0.943	0.647	0.283
trnG (UCC) intron	0.051	1.000	0.237	0.367
trnG (UCC)-trnR (UCU)	0.000	0.000	0.636	0.512
trnR (UCU)-atpA	0.209	0.993	0.851	0.439
atpA-atpF	0.000	0.000	0.403	0.484
atpF intron	0.071	0.900	0.346	0.545
atpF-atpH	0.090	1.000	0.462	0.737
atpH-atpI	0.204	0.673	0.563	0.501
atpI-rps2	0.024	1.000	0.265	0.344
rps2-rpoC2	0.000	0.000	0.299	0.303
rpoC2-rpoC1	0.000	0.000	0.198	0.229
rpoC1 intron	0.000	0.000	0.260	0.255
rpoC1-rpoB	0.000	0.000	0.219	0.857
rpoB-trnC (GCA)	0.025	0.194	0.561	0.678
trnC (GCA)-petN	0.000	0.000	0.579	0.228
petN-psbM	0.017	0.824	0.442	0.468
psbM-trnD (GUC)	0.233	0.992	0.645	0.094
trnD (GUC)-trnY (GUA)	0.000	0.000	0.600	0.578

psbZ	0.000	0.000	0.106	0.000	*trnY (GUA)-trnE (UUC)*	0.000	0.000	0.186	0.000
trnG-GCC	0.000	0.000	0.028	0.000	*trnE (UUC)-trnT (GGU)*	0.109	1.000	0.548	0.307
trnfM-CAU	0.000	0.000	0.053	0.000	*trnT (GGU)-psbD*	0.077	0.747	0.601	0.665
rps14	0.000	0.000	0.053	0.000	*psbC-trnS (UGA)*	0.004	0.000	0.308	0.273
psaB	0.000	0.000	0.048	0.000	*trnS (UGA)-psbZ*	0.379	1.000	0.569	0.635
psaA	0.000	0.000	0.051	0.009	*psbZ-trnG (GCC)*	0.012	0.833	0.676	0.362
ycf3	0.000	0.000	0.047	0.125	*trnG (GCC)-trnfM (CAU)*	0.080	1.000	0.480	0.427
trnS-GGA	0.000	0.000	0.092	0.000	*trnfM (CAU)-rps14*	0.000	0.000	0.294	0.234
rps4	0.000	0.000	0.061	0.000	*rps14-psaB*	0.000	0.000	0.160	0.150
trnT-UGU	0.000	0.000	0.000	0.000	*psaB-psaA*	0.000	0.000	0.000	0.000
trnL-UAA	0.000	0.000	0.058	0.200	*psaA-ycf3*	0.036	0.741	0.388	0.468
trnF-GAA	0.000	0.000	0.027	0.000	*ycf3 intron*	0.003	0.500	0.124	0.160
ndhJ	0.119	0.984	0.167	0.700	*ycf3 intron*	0.000	0.000	0.175	0.445
ndhK	0.001	0.000	0.066	0.000	*ycf3-trnS (GGA)*	0.000	0.000	0.389	0.109
ndhC	0.000	0.000	0.063	0.000	*trnS (GGA)-rps4*	0.007	0.000	0.380	0.266
trnV-UAC	0.000	0.000	0.105	0.250	*rps4-trnT (UGU)*	0.247	0.989	0.598	0.269
trnM-CAU	0.000	0.000	0.014	0.000	*trnT (UGU)-trnL (UAA)*	0.067	0.917	0.655	0.314

atpE	0.000	0.000	0.109	0.044	***trnL (UAA) intron***	0.002	0.000	0.478	0.318
atpB	0.000	0.000	0.061	0.000	***trnL (UAA)-trnF (GAA)***	0.000	0.000	0.831	0.859
rbcL	0.000	0.000	0.085	0.203	***trnF (GAA)-ndhJ***	0.297	1.000	0.641	0.668
accD	0.087	0.993	0.402	0.503	***ndhJ-ndhK***	0.000	0.000	0.374	0.449
psaI	0.000	0.000	0.145	0.176	***ndhK-ndhC***	0.000	0.000	0.068	0.667
ycf4	0.000	0.000	0.076	0.071	***ndhC-trnV (UAC)***	0.195	0.671	0.722	0.429
cemA	0.000	0.000	0.091	0.000	***trnV (UAC) intron***	0.002	0.000	0.136	0.259
petA	0.000	0.000	0.091	0.068	***trnV (UAC)-trnM (CAU)***	0.000	0.000	0.253	0.521
psbJ	0.000	0.000	0.098	0.000	***trnM (CAU)-atpE***	0.000	0.000	0.380	0.171
psbL	0.000	0.000	0.017	0.000	***atpB-rbcL***	0.001	1.000	0.207	0.250
psbF	0.000	0.000	0.058	0.000	***rbcL-accD***	0.000	0.000	0.313	0.363
psbE	0.000	0.000	0.012	0.000	***accD-psaI***	0.018	1.000	0.356	0.158
petL	0.000	0.000	0.125	0.000	***psaI-ycf4***	0.000	0.000	0.259	0.353
petG	0.000	0.000	0.061	0.000	***ycf4-cemA***	0.000	0.000	0.558	0.249
trnW-CCA	0.000	0.000	0.068	0.000	***cemA-petA***	0.005	0.000	0.197	0.026
trnP-UGG	0.000	0.000	0.014	0.000	***petA-psbJ***	0.028	0.036	0.376	0.323
psaJ	0.000	0.000	0.185	0.000	***psbJ-psbL***	0.000	0.000	0.062	0.000
rpl33	0.000	0.000	0.106	0.273	***psbL-psbF***	0.000	0.000	0.045	0.000
rps18	0.003	0.000	0.095	0.300	***psbF-psbE***	0.000	0.000	0.111	0.000
rpl20	0.000	0.000	0.079	0.000	***psbE-petL***	0.056	0.863	0.430	0.252
rps12	0.000	0.000	0.044	0.000	***petL-petG***	0.000	0.000	0.226	0.091

	clpP	0.002	0.000	0.152	0.000	***petG-trnW (CCA)***	0.008	1.000	0.384	0.188
	psbB	0.001	0.000	0.065	0.020	***trnW (CCA)-trnP (UGG)***	0.000	0.000	0.364	0.437
	psbT	0.000	0.000	0.123	0.429	***trnP (UGG)-psaJ***	0.005	1.000	0.563	0.235
	psbN	0.000	0.000	0.023	0.000	***psaJ-rpl33***	0.014	0.833	0.319	0.293
	psbH	0.000	0.000	0.081	0.000	***rpl33-rps18***	0.000	0.000	0.270	0.280
	petB	0.000	0.000	0.049	0.000	***rps18-rpl20***	0.005	0.000	0.328	0.250
	petD	0.000	0.000	0.053	0.231	***rpl20-rps12***	0.003	0.500	0.193	0.219
	rpoA	0.001	0.000	0.096	0.063	***rps12-clpP***	0.000	0.000	0.531	0.372
	rps11	0.000	0.000	0.084	0.000	***clpP intron***	0.002	1.000	0.568	0.144
	rpl36	0.000	0.000	0.096	0.000	***clpP intron***	0.001	1.000	0.247	0.220
	rps8	0.000	0.000	0.091	0.000	***clpP-psbB***	0.002	0.000	0.268	0.338
	rpl14	0.000	0.000	0.035	0.000	***psbB-psbT***	0.063	0.333	0.565	0.338
	rpl16	0.000	0.000	0.098	0.000	***psbT-psbN***	0.000	0.000	0.117	0.000
	rps3	0.000	0.000	0.114	0.000	***psbN-psbH***	0.000	0.000	0.158	0.111
	rpl22	0.002	0.000	0.252	0.287	***psbH-petB***	0.000	0.000	0.199	0.407
IRb	***rps19***	0.007	0.000	0.188	0.109	***petB intron***	0.000	0.000	0.220	0.261
	rpl2	0.001	0.000	0.012	0.000	***petB-petD***	0.000	0.000	0.208	0.349
	rpl23	0.000	0.000	0.018	0.000	***petD intron***	0.001	0.000	0.232	0.253
	trnI-CAU	0.000	0.000	0.000	0.000	***petD-rpoA***	0.000	0.000	0.309	0.275
	ycf2	0.039	1.000	0.239	0.101	***rpoA-rps11***	0.000	0.000	0.059	0.250
	trnL-CAA	0.160	1.000	0.160	0.000	***rps11-rpl36***	0.000	0.000	0.239	0.303
	ndhB	0.000	0.000	0.012	0.000	***rpl36-rps8***	0.004	0.500	0.375	0.161
	rps7	0.000	0.000	0.011	0.000	***rps8-rpl14***	0.000	0.000	0.307	0.333
	rps12	0.000	0.000	0.004	0.000	***rpl14-rpl16***	0.007	0.000	0.810	0.832

	orf70	0.000	0.000	0.026	0.000		***rpl16 intron***	0.002	0.500	0.341	0.239
	trnV-GAC	0.000	0.000	0.028	0.000		***rpl16-rps3***	0.000	0.000	0.233	0.176
	rrn16	0.000	0.000	0.003	0.000		***rpl22-rps19***	0.000	0.000	0.472	0.571
	trnI-GAU	0.000	0.000	0.014	0.000	IRb	***rps19-rpl2***	0.000	0.000	0.585	0.263
	trnA-UGC	0.000	0.000	0.000	0.000		***rpl2 intron***	0.000	0.000	0.044	0.667
	rrn23	0.000	0.000	0.007	0.000		***rpl2-rpl23***	0.000	0.000	0.000	0.000
	rrn4.5	0.000	0.000	0.010	0.000		***rpl23-trnI (CAU)***	0.000	0.000	0.031	0.000
	rrn5	0.000	0.000	0.000	0.000		***trnI (CAU)-ycf2***	0.000	0.000	0.108	0.500
	trnR-ACG	0.000	0.000	0.000	0.000		***ycf2-trnL (CAA)***	0.426	1.000	0.700	0.131
	trnN-GUU	0.000	0.000	0.055	0.000		***trnL (CAA)-ndhB***	0.000	0.000	0.054	0.000
SSC	***ycf1***	0.016	0.900	0.323	0.083		***ndhB intron***	0.000	0.000	0.016	0.000
	ndhF	0.011	0.720	0.176	0.082		***ndhB-rps7***	0.000	0.000	0.042	0.643
	rpl32	0.000	0.000	0.201	0.061		***rps7-rps12***	0.000	0.000	0.038	0.000
	trnL-UAG	0.000	0.000	0.050	0.000		***rps12 intron***	0.000	0.000	0.033	0.000
	ccsA	0.001	0.000	0.130	0.000		***rpl12-orf70***	0.008	1.000	0.186	0.237
	ndhD	0.001	0.000	0.110	0.000		***orf70-trnV (GAC)***	0.000	0.000	0.234	0.000
	psaC	0.000	0.000	0.049	0.000		***trnV (GAC)-rrn16***	0.000	0.000	0.035	0.000
	ndhE	0.000	0.000	0.069	0.000		***rrn16-trnI (GAU)***	0.000	0.000	0.030	0.111
	ndhG	0.002	0.000	0.092	0.000		***trnI (GAU) intron***	0.001	1.000	0.165	0.032

ndhI	0.002	0.000	0.095	0.140
ndhA	0.001	0.000	0.111	0.000
ndhH	0.001	0.000	0.077	0.000
rps15	0.004	0.000	0.121	0.000
ycf1	0.003	0.563	0.430	0.312

	trnI (GAU)-trnA (UGC)	0.000	0.000	0.137	0.100
	trnA (UGC) intron	0.000	0.000	0.012	0.000
	trnA (UGC)-rrn23	0.000	0.000	0.013	0.000
	rrn23-rrn4.5	0.000	0.000	0.000	0.000
	rrn4.5-rrn5	0.000	0.000	0.158	0.167
	rrn5-trnR (ACG)	0.000	0.000	0.195	0.164
	trnR (ACG)-trnN (GUU)	0.006	0.000	0.099	0.333
	trnN (GUU)-ycf1	0.000	0.000	0.192	0.708
SSC	***ndhF-rpl32***	0.015	0.800	0.561	0.201
	rpl32-trnL (UAG)	0.239	0.758	0.839	0.473
	trnL (UAG)-ccsA	0.000	0.000	0.614	0.532
	ccsA-ndhD	0.084	1.000	0.641	0.228
	ndhD-psaC	0.000	0.000	0.273	0.139
	psaC-ndhE	0.000	0.000	0.291	0.171
	ndhE-ndhG	0.000	0.000	0.278	0.294
	ndhG-ndhI	0.009	0.000	0.287	0.245
	ndhI-ndhA	0.000	0.000	0.125	0.000
	ndhA intron	0.014	0.000	0.892	0.000
	ndhA-ndhH	0.000	0.000	0.000	0.000
	ndhH-rps15	0.000	0.000	0.223	0.000
	rps15-ycf1	0.007	0.000	0.429	0.000

Note: Numbers in red represented the top-four great-variable regions for CDS and CNS of each pattern.

Table S4. List of tandem repeats in the chloroplast genome of eleven Cucurbitales species.

Species	No.	Indices		Copy Number	Consensus Size	Percent Matches	Percent Indels	Species	No.	Indices		Copy Number	Consensus Size	Percent Matches	Percent Indels
***G. cissiformis var. cissiformis**	1	9215	9737	2	260	100	0	**C. grandis**	1	10296	10358	3.8	17	91	2
	2	10265	10336	2	36	97	2		2	10295	10388	2.9	33	84	7
	3	10885	11157	2	138	100	0		3	10342	10405	2.2	29	97	2
	4	13891	14011	2.1	57	98	1		4	34265	34323	2.4	25	88	5
	5	15249	15495	2	123	100	0		5	34265	34315	2	26	92	3
	6	28622	28842	2	110	100	0		6	93944	94000	3.2	18	94	0
	7	32186	32443	2	128	100	0		7	110025	110090	2.1	32	97	0
	8	32634	32898	2	131	100	0		8	133695	133760	2.1	32	97	0
	9	34123	34287	2	82	100	0		9	149785	149841	3.2	18	94	0
	10	34692	34803	2	55	100	0	**C. sativus**	1	8908	8969	2.9	22	90	7
	11	38417	38576	2.1	78	100	0		2	10756	10801	2	23	100	0
	12	45349	45547	2	99	100	0		3	32981	33023	2	22	100	0
	13	49351	49462	2	55	100	0		4	50099	50152	3.1	17	89	2
	14	51345	51780	2	218	98	0		5	60640	60749	4	27	86	1
	15	52097	52224	2	63	100	0		6	93000	93052	2.9	18	94	0
	16	54012	54071	1.9	31	96	0		7	109106	109171	2.1	32	97	0
	17	63404	63873	2	235	100	0		8	110772	110815	2	22	100	0
	18	67827	68195	2	182	100	0		9	115003	115064	7	9	82	10
	19	68295	68354	3.8	15	83	12		10	129093	129268	29.3	6	70	21
	20	68291	68358	2.1	33	100	0		11	129097	129232	5.7	24	73	19

	21	76854	77057	1.9	110	100	0
	22	79792	79857	2.4	28	95	5
	23	94431	94487	3.2	18	94	0
	24	96794	96909	2	58	100	0
	25	110403	110468	2.1	32	97	0
	26	116651	117124	2	234	100	0
	27	118446	118661	2	107	100	0
	28	123707	123822	2	57	100	0
	29	134107	134172	2.1	32	97	0
	30	147665	147780	2	58	100	0
	31	150087	150143	3.2	18	94	0
****G. cissiformis var. villosa***	1	9435	9917	1.9	259	98	0
	2	13912	14137	2.1	110	100	0
	3	28653	28866	2	106	100	0
	4	38064	38273	2	106	100	0
	5	44945	45120	2	87	100	0
	6	53231	53321	2.9	31	98	0
	7	62814	63283	2	235	100	0
	8	67183	67613	2	212	99	0
	9	76243	76456	2	106	100	0
	10	79193	79258	2.4	28	95	5
	11	93832	93888	3.2	18	94	0
	12	109722	109787	2.1	32	97	0
	12	129097	129280	5.9	30	76	16
	13	129097	129341	7.1	35	74	18
	14	129139	129262	2.3	53	84	8
	15	129241	129397	13.6	12	82	7
	16	129133	129383	8.6	30	80	11
	17	131169	131212	2	22	100	0
	18	132811	132876	2.1	32	97	0
	19	148930	148982	2.9	18	94	0
C. moschata	1	51196	52160	9.8	95	84	7
	2	51196	52160	4.9	192	81	9
	3	61843	61987	12.1	12	93	5
	4	95429	95593	9.2	18	97	0
	5	111263	111328	2.1	32	97	0
	6	131078	131142	3.1	21	95	0
	7	131229	131293	2.7	24	85	4
	8	134660	134725	2.1	32	97	0
	9	150395	150559	9.2	18	97	0
M. charantia	1	46220	46281	3.9	16	100	0
	2	72263	72311	2	24	100	0
	3	94039	94095	3.2	18	94	0
	4	110137	110202	2.1	32	97	0
	5	133787	133852	2.1	32	97	0
	6	149894	149950	3.2	18	94	0

	13	115940	116451	2	251	99	0
	14	117755	118019	2	131	100	0
	15	123060	123145	2	42	100	0
	16	133441	133506	2.1	32	97	0
	17	149340	149396	3.2	18	94	0
***H. lijiangensis**	1	4428	4726	2	148	100	0
	2	28072	28277	2.1	100	98	0
	3	32059	32341	2	140	100	0
	4	33289	33470	2	92	100	0
	5	34916	35117	2	101	100	0
	6	44768	44891	2.2	57	94	4
	7	45051	45182	2	66	100	0
	8	49270	50027	2	378	100	0
	9	51324	51365	2	21	100	0
	10	51846	51894	2	24	100	0
	11	53778	54271	2	246	100	0
	12	54298	54456	2.6	64	94	5
	13	63609	64067	2	228	100	0
	14	68127	68485	2	178	100	0
	15	76981	77196	2	107	100	0
	16	92119	92170	2.5	21	90	0
	17	96902	97068	2.1	81	100	0
	18	110743	110808	2.1	32	97	0
	19	117267	117529	2	131	100	0

L. siceraria	1	4569	4611	2	21	100	0
	2	6777	6823	2	23	100	0
	3	31318	31361	2.8	16	100	0
	4	54307	54348	2.1	20	100	0
	5	60935	61075	3.9	36	92	1
	6	61323	61370	2	24	100	0
	7	93069	93136	3.2	21	82	0
	8	95495	95551	3.2	18	94	0
	9	111724	111789	2.1	32	97	0
	10	131369	131531	4.5	36	99	0
	11	135430	135495	2.1	32	97	0
	12	151668	151724	3.2	18	94	0
	13	154084	154172	4.1	21	81	5
C. laevigata	1	2025	2066	3.5	12	100	0
	2	9614	9683	2.1	33	82	10
	3	9659	9716	1.9	30	86	10
	4	10961	11023	2.5	26	86	2
	5	11685	11739	2.4	23	96	3
	6	16541	16580	2.1	19	100	0
	7	18518	18561	2	22	100	0
	8	33642	33694	2.8	20	91	5
	9	39647	39715	2.3	30	87	7
	10	49806	49847	2	21	100	0
	11	50475	50518	2	22	100	0

	20	117951	118026	2.4	32	100	0
	21	119054	119307	2	125	100	0
	22	134830	134895	2.1	32	97	0
	23	148570	148736	2.1	81	100	0
	24	153468	153519	2.5	21	90	0
G. pentaphyllum	1	10350	10420	2.5	30	84	11
	2	44537	44592	2.5	23	85	14
	3	91514	91596	3.8	22	84	7
	4	91536	91603	3.2	21	87	0
	5	93955	94009	3.1	18	89	0
	6	101919	101963	2.1	22	95	4
	7	152732	152820	4.1	21	84	5
C. lanatus	1	10579	10641	3.8	17	91	4
	2	10579	10641	1.9	33	87	6
	3	93965	94021	3.2	18	94	0
	4	110063	110128	2.1	32	97	0
	5	133624	133689	2.1	32	97	0
	6	149731	149787	3.2	18	94	0
	12	55637	55693	3.2	19	82	14
	13	71704	71768	3	22	93	2
	14	71976	72021	2	23	100	0
	15	93746	93813	3.2	21	82	0
	16	96181	96273	5.2	18	93	0
	17	107705	107744	2	20	100	0
	18	111848	111913	2.1	32	97	0
	19	136152	136217	2.1	32	97	0
	20	140321	140360	2	20	100	0
	21	151792	151884	5.2	18	93	0
	22	154253	154341	4.1	21	81	5

Table S5. List of dispersed repeats and palindromic repeats in the chloroplast genome of eleven Cucurbitales species.

Species	Repeat	Length of the 1st repeat	Starting position of the 1st repeat	Direction	Length of the 2nd repeat	Starting position of the 2nd repeat	Mismatch	Species	Repeat	Length of the 1st repeat	Starting position of the 1st repeat	Direction	Length of the 2nd repeat	Starting position of the 2nd repeat	Mismatch
**_*G. cissiformis var. cissiformis_**	**Dispersed repeats**	30	10433	F	30	39514	-2	***C. sativus***	**Dispersed repeats**	39	100354	F	39	121920	-1
		30	41727	F	30	43951	-2			41	129344	F	41	129356	-1
		31	10264	F	31	10300	0			44	129092	F	44	129134	-1
		31	79791	F	31	79819	0			44	129092	F	44	129224	-2
		31	54011	F	31	54042	-2			45	129133	F	45	129187	-1
		34	110402	F	34	110434	-1			45	129187	F	45	129223	-2
		34	134106	F	34	134138	-1			45	129274	F	45	129352	-2
		35	68290	F	35	68323	0			46	129162	F	46	129192	-2
		37	46749	F	37	101503	-3			46	129096	F	46	129162	-3
		37	46749	F	37	123370	-3			47	129284	F	47	129350	-1
		38	41719	F	38	43943	-3			51	129274	F	51	129310	-3
		39	94430	F	39	94448	-2			52	129096	F	52	129192	-1
		39	150086	F	39	150104	-2			53	129296	F	53	129320	-3
		41	101502	F	41	123369	0			55	129302	F	55	129344	-1
		47	51515	F	47	51733	-3			55	129314	F	55	129344	-3
		57	34691	F	57	34746	0			59	129284	F	59	129338	-1

	57	49350	F	57	49405	0
	58	96793	F	58	96851	0
	58	147664	F	58	147722	0
	59	123706	F	59	123763	0
	60	13894	F	60	13951	0
	65	52096	F	65	52159	0
	82	38416	F	82	38494	0
	83	34122	F	83	34204	0
	94	76853	F	94	76963	0
	100	45348	F	100	45447	0
	109	118445	F	109	118552	0
	111	28621	F	111	28731	0
	124	15248	F	124	15371	0
	130	32185	F	130	32313	0
	134	32633	F	134	32764	0
	135	10884	F	135	11022	0
	187	67826	F	187	68008	0
	205	51344	F	205	51562	-2
	235	63403	F	235	63638	0
	240	116650	F	240	116884	0
	263	9214	F	263	9474	0
**Palindromic repeats**	30	8810	P	30	48196	0
	34	110402	P	34	134106	-1
	34	110434	P	34	134138	-1
	60	129126	F	60	129216	-1
	63	129274	F	63	129298	-3
	73	129284	F	73	129326	-3
	89	129296	F	89	129308	-2
	101	129284	F	101	129296	-3
**Palindromic repeats**	39	121920	P	39	141592	-1
	42	4521	P	42	4521	0
	42	120569	P	42	120569	0
	44	76532	P	44	76532	0
	45	106417	P	45	135520	0
	48	111442	P	48	130493	-1
	76	94651	P	76	147260	-2
	90	106326	P	90	135567	0
	133	94594	P	133	147260	-3
	149	94730	P	149	147108	0
	158	111723	P	158	130104	0
	179	94700	P	179	147108	-2
	185	94514	P	185	147288	-3
	213	94666	P	213	147108	-3
	234	111490	P	234	130260	0
	306	105009	P	306	136670	0
	316	111174	P	316	130493	-2
	516	110657	P	516	130810	0
	550	104460	P	550	136974	0

		41	123369	P	41	143030	0
		58	96793	P	58	147664	0
		58	96851	P	58	147722	0
		7596	105684	P	7596	131294	0
G. cissiformis							
var. villosa	**Dispersed repeats**	30	10647	F	30	39113	-2
		30	41311	F	30	43535	-2
		30	112015	F	30	131182	-2
		30	23944	F	30	83953	-3
		31	79192	F	31	79220	0
		31	53230	F	31	53292	-2
		32	9060	F	32	37866	-3
		34	109721	F	34	109753	-1
		34	133440	F	34	133472	-1
		37	46330	F	37	100829	-3
		37	46330	F	37	122710	-3
		38	41303	F	38	43527	-3
		39	93831	F	39	93849	-2
		39	149339	F	39	149357	-2
		41	100828	F	41	122709	0
		41	9655	F	41	9947	-1
		44	123059	F	44	123101	0
		60	53230	F	60	53261	-1
		89	44944	F	89	45031	0
		104	38063	F	104	38169	0
		628	110545	P	628	130810	-1
		1011	109534	P	1011	131437	0
		1016	105312	P	1016	135656	0
		1302	103157	P	1302	137525	0
		1489	86688	P	1489	153804	0
		1598	101558	P	1598	138828	0
		3074	106461	P	3074	132446	0
		6283	88178	P	6283	147520	0
		6405	95153	P	6405	140427	0
C.	**Dispersed**						
moschata	**repeats**	73	51495	F	73	51992	-2
		76	51593	F	76	51992	-3
		78	95461	F	78	95515	-1
		78	95443	F	78	95515	-3
		82	51610	F	82	51717	-3
		84	51734	F	84	51829	-3
		84	51734	F	84	51931	-3
		86	95428	F	86	95500	-3
		86	150401	F	86	150473	-3
		87	51827	F	87	51929	0
		92	51259	F	92	51754	-2
		96	95461	F	96	95497	-1
		96	95443	F	96	95497	-3
		97	61842	F	97	61866	-3
		102	51812	F	102	51914	-2

		108	28652	F	108	28758	0
		108	76242	F	108	76348	0
		116	13911	F	116	14021	0
		134	117754	F	134	117885	0
		198	9461	F	198	9719	-2
		210	9449	F	210	9707	-3
		214	67187	F	214	67399	0
		235	62813	F	235	63048	0
	**Palindromic repeats**	30	9062	P	30	47777	0
		30	112015	P	30	112015	-2
		30	131182	P	30	131182	-2
		30	37868	P	30	47777	-3
		34	109721	P	34	133440	-1
		34	109753	P	34	133472	-1
		37	7478	P	37	7478	-3
		37	46330	P	37	142361	-3
		39	93831	P	39	149339	-2
		39	93849	P	39	149357	-2
		41	122709	P	41	142358	0
		44	76601	P	44	76601	-2
**H. lijiangensis*	**Dispersed repeats**	30	41348	F	30	43572	-2
		30	10091	F	30	39119	-3
		30	23326	F	30	84683	-3
		31	62437	F	31	62480	-1
		103	51465	F	103	51563	-1
		104	95428	F	104	95482	-3
		104	150401	F	104	150455	-3
		114	95461	F	114	95479	-1
		114	95443	F	114	95479	-3
		119	51449	F	119	51547	-2
		122	95428	F	122	95464	-3
		122	150401	F	122	150437	-3
		125	95450	F	125	95468	-2
		143	95432	F	143	95450	-3
		143	150394	F	143	150412	-3
	**Palindromic repeats**	77	0	P	77	88266	0
		78	95461	P	78	150394	-1
		78	95515	P	78	150448	-1
		78	95443	P	78	150394	-3
		78	95515	P	78	150466	-3
		86	95428	P	86	150401	-3
		86	95500	P	86	150473	-3
		96	95461	P	96	150394	-1
		96	95497	P	96	150430	-1
		96	95443	P	96	150394	-3
		96	95497	P	96	150448	-3
		104	95428	P	104	150401	-3
		104	95482	P	104	150455	-3

32	8650	F	32	37950	-3
32	51838	F	32	51862	-3
32	92120	F	32	92141	-3
32	153464	F	32	153485	-3
34	110742	F	34	110774	-1
34	134829	F	34	134861	-1
37	46373	F	37	101865	-3
37	46373	F	37	123978	-3
41	101864	F	41	123977	0
44	117950	F	44	117982	0
48	44776	F	48	44833	0
66	45050	F	66	45116	0
66	54301	F	66	54365	0
86	96901	F	86	96982	0
86	148569	F	86	148650	0
90	33288	F	90	33380	0
96	28076	F	96	28176	0
101	34915	F	101	35016	0
109	76980	F	109	77087	0
129	119053	F	129	119178	0
132	117266	F	132	117397	0
143	32058	F	143	32198	0
151	4427	F	151	4575	0
181	68126	F	181	68304	0
231	63608	F	231	63836	0

		114	95461	P	114	150394	-1
		114	95479	P	114	150412	-1
		114	95443	P	114	150394	-3
		114	95479	P	114	150430	-3
		122	95428	P	122	150401	-3
		122	95464	P	122	150437	-3
		125	95450	P	125	150394	-2
		125	95468	P	125	150412	-2
		143	95432	P	143	150394	-3
		143	95450	P	143	150412	-3
M. charantia	**Dispersed repeats**	30	41863	F	30	44087	-2
		30	114024	F	30	133164	-2
		30	6776	F	30	6799	-3
		30	10783	F	30	39605	-3
		30	46897	F	30	102833	-3
		31	85206	F	31	88294	0
		32	31313	F	32	31329	-1
		32	9162	F	32	38384	-3
		32	93068	F	32	93089	-3
		32	154097	F	32	154118	-3
		33	60934	F	33	61006	-3
		34	111723	F	34	111755	-1
		34	135429	F	34	135461	-1
		35	54306	F	35	54326	-3
		37	46885	F	37	102821	-2

		248	53777	F	248	54023	0
		380	49269	F	380	49647	0
	**Palindromic repeats**	30	8652	P	30	47837	0
		30	33919	P	30	33919	-2
		30	49015	P	30	49047	-2
		30	37952	P	30	47837	-3
		32	38920	P	32	38920	0
		32	92120	P	32	153464	-3
		32	92141	P	32	153485	-3
		33	5892	P	33	5892	-1
		33	33963	P	33	33963	-1
		33	10130	P	33	10130	-3
		34	110742	P	34	134829	-1
		34	110774	P	34	134861	-1
		37	46373	P	37	143735	-3
		39	69447	P	39	69447	-3
		41	123977	P	41	143732	0
		44	77341	P	44	77341	-2
		86	96901	P	86	148569	0
		86	96982	P	86	148650	0
G. pentaphyllum	**Dispersed repeats**	30	9951	F	30	38565	-2
		30	40803	F	30	43027	-2
		30	93954	F	30	93972	-2
		30	112254	F	30	132049	-2
		37	46885	F	37	124481	-2
		38	61005	F	38	61041	-3
		39	102820	F	39	124480	0
		39	95494	F	39	95512	-2
		39	151667	F	39	151685	-2
		55	131368	F	55	131476	-1
		59	60934	F	59	60970	0
		91	131368	F	91	131440	-1
		127	131368	F	127	131404	-1
	**Palindromic repeats**	30	9164	P	30	48364	0
		30	34611	P	30	34611	0
		30	123138	P	30	123138	0
		30	114024	P	30	114024	-2
		30	133164	P	30	133164	-2
		30	38386	P	30	48364	-3
		30	46897	P	30	144355	-3
		31	9051	P	31	51918	-3
		32	132053	P	32	132053	-2
		32	93068	P	32	154097	-3
		32	93089	P	32	154118	-3
		33	39777	P	33	39777	-3
		34	111723	P	34	135429	-1
		34	111755	P	34	135461	-1
		35	50968	P	35	50968	-3

		30	150331	F	30	150349	-2
		30	1	F	30	9975	-3
		30	23106	F	30	84119	-3
		30	45857	F	30	101293	-3
		31	61726	F	31	61769	-2
		32	91535	F	32	91556	-2
		32	152745	F	32	152766	-2
		32	8549	F	32	37420	-3
		37	45845	F	37	101281	-2
		37	45845	F	37	123357	-2
		41	101280	F	41	123356	0
	**Palindromic**						
	**repeats**	30	8551	P	30	47188	0
		30	67863	P	30	67863	0
		30	6864	P	30	6864	-2
		30	93954	P	30	150331	-2
		30	93972	P	30	150349	-2
		30	112254	P	30	112254	-2
		30	132049	P	30	132049	-2
		30	8720	P	30	8756	-3
		30	37422	P	30	47188	-3
		30	44546	P	30	44559	-3
		30	45857	P	30	143010	-3
		32	91535	P	32	152745	-2
		37	46885	P	37	144360	-2
		39	124480	P	39	144359	0
		39	95494	P	39	151667	-2
		39	95512	P	39	151685	-2
		39	4515	P	39	4515	-3
		44	78087	P	44	78087	0
		52	118300	P	52	118300	0
		264	0	P	264	84939	0
	**Dispersed**						
L. siceraria	**repeats**	30	46219	F	30	46251	0
		30	10468	F	30	38561	-2
		30	112409	F	30	131549	-2
		30	40801	F	30	43025	-3
		30	101386	F	30	123053	-3
		32	8859	F	32	37393	-3
		34	110136	F	34	110168	-1
		34	133786	F	34	133818	-1
		37	45784	F	37	101374	-1
		37	45784	F	37	123041	-2
		39	101373	F	39	123040	-1
		39	94038	F	39	94056	-2
		39	149893	F	39	149911	-2
		46	46219	F	46	46235	0
	**Palindromic**						
	**repeats**	30	8861	P	30	47324	0

		32	91556	P	32	152766	-2
		35	116330	P	35	116330	-1
		36	13489	P	36	13489	-2
		37	45845	P	37	143015	-2
		41	123356	P	41	143012	0
		44	76778	P	44	76778	0
		47	48425	P	47	48440	-3
C. lanatus	**Dispersed repeats**	30	10531	F	30	38434	-2
		30	40671	F	30	42895	-3
		30	101316	F	30	122960	-3
		32	8904	F	32	37372	-3
		34	110062	F	34	110094	-1
		34	133623	F	34	133655	-1
		37	45667	F	37	101304	-1
		37	45667	F	37	122948	-2
		39	101303	F	39	122947	-1
		39	93964	F	39	93982	-2
		39	149730	F	39	149748	-2
	**Palindromic repeats**	30	8906	P	30	47172	0
		30	37374	P	30	47172	-3
		30	122960	P	30	142405	-3
		33	65413	P	33	65413	-1
		34	110062	P	34	133623	-1
		34	110094	P	34	133655	-1

		30	112409	P	30	112409	-2
		30	131549	P	30	131549	-2
		30	37395	P	30	47324	-3
		30	123053	P	30	142572	-3
		33	65459	P	33	65459	-1
		34	110136	P	34	133786	-1
		34	110168	P	34	133818	-1
		35	116410	P	35	116410	-3
		36	33893	P	36	33893	0
		37	45784	P	37	142577	-1
		39	123040	P	39	142576	-1
		39	94038	P	39	149893	-2
		39	94056	P	39	149911	-2
		39	62200	P	39	62200	-3
		42	121695	P	42	121695	0
		43	48772	P	43	48772	-1
		43	4553	P	43	4553	-3
		44	76717	P	44	76717	-2
		48	4545	P	48	4545	-2
C. laevigata	**Dispersed repeats**	30	2024	F	30	2036	0
		30	11684	F	30	11707	-2
		31	71715	F	31	71737	-1
		31	47100	F	31	103152	-2
		31	72926	F	31	72954	-2

		36	33882	P	36	33882	0
		37	45667	P	37	142410	-1
		39	122947	P	39	142409	-1
		39	93964	P	39	149730	-2
		39	93982	P	39	149748	-2
		39	10588	P	39	10588	-3
		39	62153	P	39	62153	-3
		42	121603	P	42	121603	0
		43	4611	P	43	4611	-3
		44	76661	P	44	76661	0
		48	4603	P	48	4603	-2
		55	0	P	55	86775	0
C. grandis	**Dispersed repeats**	30	10341	F	30	10371	0
		30	10202	F	30	38445	-2
		30	112310	F	30	131444	-2
		30	40673	F	30	42897	-3
		31	4550	F	31	48960	-2
		32	4560	F	32	48959	-3
		32	8593	F	32	37277	-3
		34	110024	F	34	110056	-1
		34	133694	F	34	133726	-1
		34	5321	F	34	73121	-3
		35	10306	F	35	10323	-2
		37	45662	F	37	101269	-1
		37	45662	F	37	122915	-2
		32	151816	F	32	151852	-2
		33	151833	F	33	151851	-1
		34	111847	F	34	111879	-1
		34	136151	F	34	136183	-1
		34	63552	F	34	63618	-3
		37	47092	F	37	125255	-1
		39	96180	F	39	96234	0
		39	151791	F	39	151845	0
		39	103142	F	39	125253	-2
		39	47092	F	39	103144	-3
		41	96196	F	41	96232	-2
		41	96214	F	41	96232	-3
		50	96180	F	50	96198	-2
		50	151816	F	50	151834	-2
		51	96180	F	51	96216	-3
		51	151797	F	51	151833	-3
		53	96195	F	53	96213	-3
		53	151798	F	53	151816	-3
	**Palindromic repeats**	30	9295	P	30	48719	0
		31	47100	P	31	144881	-2
		32	132690	P	32	132690	0
		34	111847	P	34	136151	-1
		34	111879	P	34	136183	-1
		34	18364	P	34	18368	-3
		35	80176	P	35	125256	-3

	39	101268	F	39	122914	-1
	39	93943	F	39	93961	-2
	39	149784	F	39	149802	-2
**Palindromic repeats**	31	4573	P	31	48960	-2
	33	65440	P	33	65440	-1
	34	110024	P	34	133694	-1
	34	110056	P	34	133726	-1
	37	45662	P	37	142478	-1
	39	122914	P	39	142477	-1
	39	93943	P	39	149784	-2
	39	93961	P	39	149802	-2
	42	121569	P	42	121569	0
	44	76662	P	44	76662	0
	48	4553	P	48	4553	-2
	35	86370	P	35	86370	-3
	36	86394	P	36	86394	0
	39	96180	P	39	151791	0
	39	96234	P	39	151845	0
	39	125253	P	39	144883	-2
	39	47092	P	39	144881	-3
	40	11567	P	40	11567	-2
	41	96196	P	41	151791	-2
	41	96232	P	41	151827	-2
	41	96214	P	41	151791	-3
	41	96232	P	41	151809	-3
	44	78831	P	44	78831	0
	50	96180	P	50	151816	-2
	50	96198	P	50	151834	-2
	51	96180	P	51	151797	-3
	51	96216	P	51	151833	-3
	53	96195	P	53	151798	-3
	53	96213	P	53	151816	-3
	65	50169	P	65	50169	-3

Table S6. List of SSRs in the chloroplast genome of eleven Cucurbitales species.

Species	SSR nr.	SSR type	Motif	Repeat Number	Size	Start position	End position	Species	SSR nr.	SSR type	Motif	Repeat Number	Size	Start position	End position
***G. cissiformis var. cissiformis**	1	p1	A	12	12	3217	3228	***C. sativus***	1	p1	A	10	10	1653	1662
	2	p1	A	11	11	4227	4237		2	p3	TTC	4	12	3061	3072
	3	p2	TA	5	10	6404	6413		3	p1	A	12	12	3197	3208
	4	p1	T	10	10	6705	6714		4	p1	A	10	10	4257	4266
	5	p1	T	12	12	8558	8569		5	p2	TA	5	10	4538	4547
	6	p1	A	15	15	8777	8791		6	p1	A	14	14	6721	6734
	7	p1	A	10	10	12968	12977		7	p1	A	15	15	7927	7941
	8	p1	A	10	10	14584	14593		8	p1	A	15	15	8174	8188
	9	p1	T	10	10	17785	17794		9	p1	A	10	10	8485	8494
	10	p1	T	13	13	19979	19991		10	p1	A	16	16	9292	9307
	11	p2	AT	5	10	21361	21370		11	p2	AT	5	10	10301	10310
	12	p1	A	11	11	24132	24142		12	p4	AATT	3	12	10321	10332
	13	p4	TATT	3	12	24456	24467		13	p1	T	10	10	10916	10925
	14	p1	T	10	10	27682	27691		14	p2	TA	5	10	11030	11039
	15	p1	A	10	10	28344	28353		15	p1	A	10	10	14132	14141
	16	p1	T	11	11	28847	28857		16	p1	T	11	11	16334	16344
	17	p2	TA	5	10	28933	28942		17	p1	T	13	13	19442	19454
	18	p2	TA	5	10	30366	30375		18	p2	AT	5	10	20821	20830
	19	p2	TA	5	10	31147	31156		19	p1	A	16	16	23625	23640
	20	p1	T	15	15	32011	32025		20	p1	T	10	10	27193	27202
	21	p1	A	13	13	32201	32213		21	p1	A	10	10	28483	28492

22	p1	A	13	13	32329	32341
23	p2	AT	5	10	32966	32975
24	p2	TA	5	10	35206	35215
25	p3	ATA	4	12	39128	39139
26	p2	TA	5	10	39339	39348
27	p1	C	10	10	43320	43329
28	p1	T	10	10	51515	51524
29	p2	TA	5	10	53955	53964
30	p1	A	11	11	62130	62140
31	p2	TA	6	12	62224	62235
32	p1	T	10	10	67896	67905
33	p1	T	10	10	68078	68087
34	p1	T	12	12	69098	69109
35	p1	A	10	10	69712	69721
36	p1	A	12	12	71296	71307
37	p1	T	10	10	71330	71339
38	p4	TAAA	3	12	72035	72046
39	p1	T	12	12	73313	73324
40	p1	A	14	14	74056	74069
41	p1	A	10	10	77467	77476
42	p1	A	14	14	84033	84046
43	p1	A	10	10	84599	84608
44	p1	T	10	10	84828	84837
45	p1	T	12	12	101705	101716
46	p1	T	10	10	105684	105693
47	p1	A	10	10	113568	113577

22	p2	TA	6	12	28537	28548
23	p4	TTTA	3	12	28706	28717
24	p1	T	10	10	28873	28882
25	p3	ACT	4	12	29287	29298
26	p1	T	10	10	29447	29456
27	p1	A	11	11	30070	30080
28	p1	A	10	10	30147	30156
29	p3	TTA	4	12	30317	30328
30	p4	TTTA	3	12	33008	33019
31	p1	T	12	12	33858	33869
32	p5	ATAAG	3	15	34512	34526
33	p4	TAAT	3	12	38825	38836
34	p4	TAAT	3	12	38841	38852
35	p1	T	10	10	44145	44154
36	p1	A	12	12	44718	44729
37	p1	T	10	10	45046	45055
38	p1	T	10	10	45576	45585
39	p1	A	11	11	47301	47311
40	p5	TTATT	3	15	48349	48363
41	p3	TTA	4	12	49056	49067
42	p1	A	11	11	49928	49938
43	p1	T	11	11	51443	51453
44	p1	C	11	11	52060	52070
45	p1	T	11	11	54288	54298
46	p1	T	10	10	57297	57306
47	p1	T	10	10	59497	59506

	48	p1	T	12	12	114064	114075	48	p1	A	12	12	59572	59583
	49	p4	AAGT	3	12	114173	114184	49	p2	TA	5	10	59625	59634
	50	p1	T	10	10	115866	115875	50	p1	T	11	11	61403	61413
	51	p1	A	11	11	116450	116460	51	p1	T	14	14	67547	67560
	52	p1	T	11	11	118667	118677	52	p1	T	10	10	69210	69219
	53	p1	A	10	10	120115	120124	53	p1	T	18	18	72999	73016
	54	p1	A	10	10	120234	120243	54	p1	A	12	12	73018	73029
	55	p2	AT	5	10	128510	128519	55	p1	T	10	10	73793	73802
	56	p1	A	13	13	130345	130357	56	p3	TAA	4	12	74341	74352
	57	p1	A	11	11	130924	130934	57	p1	T	14	14	82386	82399
	58	p1	A	12	12	142858	142869	58	p1	A	11	11	83408	83418
G. cissiformis														
var. villosa	1	p1	A	12	12	3219	3230	59	p1	A	13	13	83434	83446
	2	p1	A	11	11	4229	4239	60	p1	A	10	10	84004	84013
	3	p2	TA	5	10	6495	6504	61	p1	T	10	10	84164	84173
	4	p3	TTA	4	12	6528	6539	62	p1	T	15	15	84819	84833
	5	p1	T	12	12	8810	8821	63	p1	T	14	14	86701	86714
	6	p1	A	16	16	9028	9043	64	p4	ATTT	4	16	89753	89768
	7	p1	A	10	10	13060	13069	65	p5	ATGAA	3	15	94825	94839
	8	p1	A	11	11	14689	14699	66	p1	T	13	13	100557	100569
	9	p1	T	10	10	17784	17793	67	p2	AG	5	10	109580	109589
	10	p1	T	13	13	19978	19990	68	p1	A	10	10	112160	112169
	11	p2	AT	5	10	21354	21363	69	p4	AAGT	3	12	112765	112776
	12	p1	A	11	11	24125	24135	70	p2	TA	5	10	114583	114592
	13	p4	TATT	3	12	24449	24460	71	p1	A	12	12	115155	115166
	14	p1	T	10	10	27675	27684	72	p1	T	13	13	115996	116008

15	p1	A	10	10	28337	28346
16	p1	T	10	10	28730	28739
17	p1	T	10	10	28836	28845
18	p2	TA	5	10	28921	28930
19	p2	TA	5	10	30355	30364
20	p2	TA	5	10	31150	31159
21	p1	T	15	15	32014	32028
22	p1	A	13	13	32204	32216
23	p2	AT	5	10	32710	32719
24	p2	TA	5	10	34795	34804
25	p3	ATA	4	12	38732	38743
26	p2	TA	5	10	38943	38952
27	p1	C	10	10	42904	42913
28	p1	T	10	10	46026	46035
29	p1	T	10	10	51052	51061
30	p4	AATA	3	12	53136	53147
31	p2	TA	5	10	53174	53183
32	p1	A	11	11	61540	61550
33	p2	TA	6	12	61634	61645
34	p1	T	11	11	67305	67315

	73	p2	AT	5	10	118751	118760
	74	p4	ATAA	4	16	119323	119338
	75	p4	CAAA	3	12	120663	120674
	76	p1	A	15	15	122232	122246
	77	p1	T	14	14	125241	125254
	78	p1	T	13	13	128277	128289
	79	p1	A	11	11	128897	128907
	80	p6	AGTCGA	3	18	129093	129110
	81	p6	GAAGTC	3	18	129133	129150
	82	p6	GAAGTC	3	18	129223	129240
	83	p6	AGTAGA	3	18	129285	129302
	84	p2	CT	5	10	132394	132403
	85	p1	A	13	13	141417	141429
	86	p5	TTTCA	3	15	147148	147162
	87	p4	AAAT	4	16	152214	152229
	88	p1	A	14	14	155268	155281
C. moschata	1	p2	TA	5	10	92	101
	2	p1	T	15	15	1622	1636
	3	p4	AATT	3	12	1949	1960
	4	p1	A	11	11	3318	3328

35	p1	T	11	11	67517	67527
36	p1	T	13	13	68504	68516
37	p1	A	10	10	69119	69128
38	p1	T	10	10	69171	69180
39	p1	T	12	12	69551	69562
40	p1	A	12	12	70698	70709
41	p4	TAAA	3	12	71437	71448
42	p1	T	13	13	72716	72728
43	p1	A	15	15	73460	73474
44	p1	A	10	10	76868	76877
45	p1	A	14	14	83435	83448
46	p1	A	10	10	84001	84010
47	p1	T	11	11	101031	101041
48	p1	A	10	10	112905	112914
49	p1	T	12	12	113401	113412
50	p4	AAGT	3	12	113510	113521
51	p1	T	10	10	115210	115219
52	p1	A	10	10	115793	115802
53	p1	A	10	10	116002	116011
54	p1	A	10	10	116253	116262
55	p1	T	11	11	117876	117886
56	p1	T	11	11	118007	118017
57	p1	A	10	10	119455	119464
58	p1	A	10	10	119574	119583
59	p4	AAAT	3	12	121234	121245
60	p2	AT	5	10	127835	127844

5	p1	T	10	10	4883	4892
6	p1	A	12	12	5039	5050
7	p1	C	10	10	5448	5457
8	p1	A	11	11	5620	5630
9	p2	GA	5	10	6583	6592
10	p1	A	11	11	6787	6797
11	p1	T	11	11	7351	7361
12	p1	A	12	12	7463	7474
13	p1	A	16	16	8139	8154
14	p1	A	17	17	8694	8710
15	p1	T	10	10	10408	10417
16	p1	A	16	16	10820	10835
17	p1	T	14	14	13025	13038
18	p1	A	12	12	13050	13061
19	p1	T	11	11	14444	14454
20	p1	A	11	11	14509	14519
21	p1	T	10	10	15582	15591
22	p1	A	10	10	15906	15915
23	p2	AT	5	10	21212	21221
24	p1	T	10	10	27551	27560
25	p2	AT	5	10	28764	28773
26	p1	A	12	12	28851	28862
27	p4	TTTA	3	12	29069	29080
28	p1	A	10	10	29503	29512
29	p1	A	15	15	30504	30518
30	p6	TAAACT	3	18	31905	31922

	61	p1	A	11	11	129679	129689	31	p1	T	10	10	34239	34248
	62	p1	A	11	11	130258	130268	32	p1	A	12	12	34598	34609
	63	p1	A	11	11	142187	142197	33	p2	TA	5	10	38764	38773
***H. lijiangensis**	1	p2	TA	5	10	19	28	34	p1	T	10	10	44587	44596
	2	p1	A	10	10	5135	5144	35	p1	T	10	10	44840	44849
	3	p1	T	10	10	8400	8409	36	p1	A	11	11	45165	45175
	4	p4	TAAA	3	12	9066	9077	37	p1	T	10	10	46051	46060
	5	p2	AT	6	12	10133	10144	38	p1	A	17	17	47040	47056
	6	p1	T	13	13	10611	10623	39	p1	A	11	11	47746	47756
	7	p1	A	10	10	12344	12353	40	p1	T	12	12	49554	49565
	8	p1	T	10	10	13060	13069	41	p2	TA	7	14	49688	49701
	9	p1	T	10	10	14952	14961	42	p1	C	12	12	53392	53403
	10	p1	T	13	13	19360	19372	43	p1	T	11	11	58747	58757
	11	p2	AT	5	10	20736	20745	44	p1	T	15	15	58826	58840
	12	p4	TATT	3	12	23844	23855	45	p1	A	12	12	58881	58892
	13	p1	T	10	10	27070	27079	46	p1	T	10	10	60945	60954
	14	p1	A	11	11	27733	27743	47	p1	T	12	12	63654	63665
	15	p2	TA	5	10	28304	28313	48	p3	AAC	4	12	70376	70387
	16	p1	A	10	10	29510	29519	49	p1	A	10	10	72997	73006
	17	p4	AAAG	3	12	29719	29730	50	p1	T	12	12	74304	74315
	18	p1	T	10	10	32829	32838	51	p1	T	11	11	75436	75446
	19	p2	TA	6	12	34420	34431	52	p1	T	12	12	84035	84046
	20	p1	G	11	11	36863	36873	53	p1	A	10	10	85057	85066
	21	p5	AATAA	3	15	37913	37927	54	p4	ATTT	4	16	92195	92210
	22	p2	TA	5	10	38172	38181	55	p1	T	11	11	102711	102721

	23	p1	C	10	10	42941	42950
	24	p1	A	10	10	45191	45200
	25	p1	T	10	10	46037	46046
	26	p1	A	13	13	47032	47044
	27	p1	A	10	10	49520	49529
	28	p1	A	10	10	49898	49907
	29	p1	A	10	10	50354	50363
	30	p2	TA	5	10	51372	51381
	31	p2	AG	6	12	53814	53825
	32	p2	AG	6	12	54060	54071
	33	p3	ATA	4	12	54467	54478
	34	p1	T	10	10	57413	57422
	35	p1	A	10	10	59798	59807
	36	p1	T	11	11	60062	60072
	37	p1	T	10	10	60187	60196
	38	p2	TA	6	12	62358	62369
	39	p1	T	10	10	62669	62678
	40	p1	T	10	10	66657	66666
	41	p1	A	11	11	69888	69898
	42	p1	T	10	10	70277	70286
	43	p1	A	12	12	72168	72179
	44	p1	A	10	10	74166	74175
	45	p1	T	11	11	77037	77047
	46	p1	T	11	11	77144	77154
	56	p1	A	13	13	111644	111656
	57	p2	AG	5	10	111736	111745
	58	p1	A	10	10	114194	114203
	59	p4	AAGT	3	12	114799	114810
	60	p3	TTA	4	12	116435	116446
	61	p1	T	10	10	116614	116623
	62	p2	AT	6	12	120756	120767
	63	p1	A	10	10	121716	121725
	64	p4	CAAA	3	12	122662	122673
	65	p1	T	12	12	124371	124382
	66	p1	T	10	10	128695	128704
	67	p2	AT	5	10	129119	129128
	68	p1	T	11	11	129712	129722
	69	p2	CT	5	10	134243	134252
	70	p1	T	13	13	134332	134344
	71	p1	A	11	11	143267	143277
	72	p4	AAAT	4	16	153778	153793
M. charantia	1	p1	A	10	10	149	158
	2	p1	T	10	10	1527	1536
	3	p1	A	14	14	3202	3215
	4	p5	GACTT	3	15	3929	3943
	5	p4	TATT	3	12	4512	4523
	6	p4	AATA	3	12	4859	4870
	7	p1	T	16	16	5177	5192

	47	p1	A	10	10	77608	77617
	48	p1	T	11	11	84190	84200
	49	p1	A	10	10	84729	84738
	50	p1	T	10	10	106032	106041
	51	p1	A	10	10	111123	111132
	52	p2	AG	5	10	111226	111235
	53	p1	A	10	10	113881	113890
	54	p4	AAGT	3	12	114486	114497
	55	p1	A	10	10	120714	120723
	56	p4	AAAT	3	12	122486	122497
	57	p4	CAAA	3	12	122721	122732
	58	p3	TAA	4	12	129670	129681
	59	p1	A	11	11	130927	130937
	60	p1	A	10	10	131666	131675
	61	p2	CT	5	10	134403	134412
	62	p1	T	10	10	134506	134515
	63	p1	A	10	10	139597	139606
G. pentaphyllum	1	p2	TA	6	12	18	29
	2	p5	AAGTA	3	15	141	155
	3	p1	A	10	10	369	378
	4	p4	TTTA	3	12	1732	1743
	5	p2	TA	6	12	4959	4970
	6	p5	GAGGG	3	15	5411	5425
	7	p1	A	11	11	5912	5922
	8	p2	TA	5	10	6558	6567
	9	p1	A	11	11	6877	6887
	10	p1	A	15	15	7684	7698
	11	p1	A	10	10	8363	8372
	12	p1	T	14	14	8915	8928
	13	p1	A	16	16	9575	9590
	14	p1	A	10	10	11044	11053
	15	p1	T	17	17	11346	11362
	16	p1	T	11	11	13317	13327
	17	p1	A	12	12	13329	13340
	18	p1	A	10	10	13957	13966
	19	p1	A	10	10	14233	14242
	20	p1	T	10	10	14722	14731
	21	p1	A	10	10	16365	16374
	22	p1	T	13	13	20290	20302
	23	p2	AT	5	10	21669	21678
	24	p1	T	10	10	27992	28001
	25	p1	A	11	11	29069	29079
	26	p1	A	10	10	30072	30081
	27	p1	T	10	10	30740	30749
	28	p1	A	10	10	32172	32181
	29	p1	T	10	10	33102	33111
	30	p3	ATA	4	12	39222	39233
	31	p1	A	10	10	39552	39561

8	p5	TTTAG	3	15	6075	6089
9	p1	A	10	10	7136	7145
10	p1	A	12	12	8308	8319
11	p4	ATAA	3	12	8933	8944
12	p2	TA	5	10	9992	10001
13	p2	AT	5	10	10350	10359
14	p2	TA	6	12	10378	10389
15	p1	T	12	12	12097	12108
16	p1	T	13	13	12825	12837
17	p1	A	13	13	12940	12952
18	p1	T	11	11	19138	19148
19	p2	AT	5	10	20515	20524
20	p1	A	11	11	23294	23304
21	p1	T	10	10	26825	26834
22	p1	A	10	10	27910	27919
23	p2	TA	5	10	28043	28052
24	p1	T	10	10	28767	28776
25	p1	A	10	10	29900	29909
26	p3	TAT	4	12	29916	29927
27	p1	T	10	10	30279	30288
28	p1	A	10	10	38711	38720
29	p1	T	10	10	45541	45550
30	p1	A	12	12	47065	47076
31	p4	TAAA	3	12	49101	49112
32	p2	AT	7	14	49913	49926
33	p2	TA	5	10	50747	50756
32	p1	A	10	10	39842	39851
33	p1	A	15	15	45688	45702
34	p1	T	10	10	46548	46557
35	p1	T	14	14	46581	46594
36	p1	T	11	11	47435	47445
37	p1	A	15	15	47547	47561
38	p1	A	11	11	48200	48210
39	p3	AAT	4	12	50162	50173
40	p4	AATA	3	12	50222	50233
41	p4	GAAT	3	12	50863	50874
42	p1	C	12	12	52914	52925
43	p3	AAT	4	12	54905	54916
44	p1	T	10	10	55978	55987
45	p1	T	10	10	57873	57882
46	p1	T	10	10	58352	58361
47	p1	A	12	12	60599	60610
48	p5	AAATT	3	15	68665	68679
49	p1	T	12	12	69137	69148
50	p1	T	10	10	70424	70433
51	p1	A	13	13	72849	72861
52	p4	TAAA	3	12	72873	72884
53	p1	T	10	10	74160	74169
54	p1	T	11	11	74241	74251
55	p1	A	11	11	74441	74451
56	p1	A	10	10	74550	74559
57	p1	T	10	10	75310	75319

34	p4	TTTA	4	16	51137	51152
35	p1	T	10	10	52546	52555
36	p1	A	10	10	53626	53635
37	p3	TAA	4	12	53683	53694
38	p1	T	10	10	56711	56720
39	p1	A	12	12	59430	59441
40	p1	T	10	10	59493	59502
41	p2	TA	5	10	61657	61666
42	p1	T	13	13	61957	61969
43	p3	AAT	4	12	65581	65592
44	p2	TA	5	10	65783	65792
45	p1	T	15	15	65960	65974
46	p1	A	10	10	66647	66656
47	p2	AT	5	10	67866	67875
48	p2	TA	5	10	67881	67890
49	p1	A	11	11	73162	73172
50	p1	T	11	11	74024	74034
51	p1	T	10	10	76582	76591
52	p1	A	13	13	83601	83613
53	p2	TC	5	10	84820	84829
54	p3	TCT	4	12	86010	86021
55	p4	ATTT	4	16	90689	90704
56	p2	AG	5	10	110463	110472
57	p1	A	10	10	113123	113132
58	p4	AAGT	3	12	113728	113739
59	p2	AT	5	10	115504	115513
58	p1	A	10	10	78355	78364
59	p1	T	11	11	78704	78714
60	p1	A	11	11	84911	84921
61	p1	T	10	10	85046	85055
62	p1	T	12	12	85226	85237
63	p1	T	14	14	86460	86473
64	p1	T	13	13	88314	88326
65	p1	T	11	11	103023	103033
66	p2	AG	5	10	112214	112223
67	p5	ATCAT	3	15	113364	113378
68	p1	A	11	11	114880	114890
69	p4	AAGT	3	12	115485	115496
70	p1	T	15	15	117288	117302
71	p2	TA	7	14	117311	117324
72	p1	A	11	11	117787	117797
73	p1	A	10	10	118083	118092
74	p1	T	13	13	118104	118116
75	p1	A	11	11	122277	122287
76	p5	AGAGT	3	15	123119	123133
77	p1	T	11	11	124938	124948
78	p1	T	11	11	127815	127825
79	p1	T	11	11	129733	129743
80	p1	T	10	10	130260	130269
81	p1	A	11	11	131630	131640
82	p5	ATATG	3	15	133839	133853
83	p2	CT	5	10	134996	135005

	60	p4	AATA	3	12	115599	115610
	61	p3	ATT	4	12	115920	115931
	62	p3	AAT	4	12	115957	115968
	63	p4	CAAA	3	12	122090	122101
	64	p1	T	13	13	128140	128152
	65	p1	T	12	12	129279	129290
	66	p1	A	16	16	130391	130406
	67	p1	A	11	11	131123	131133
	68	p2	CT	5	10	133862	133871
	69	p4	AAAT	4	16	153630	153645
C. lanatus	1	p2	TA	5	10	4623	4632
	2	p3	TAA	4	12	4704	4715
	3	p1	A	15	15	5629	5643
	4	p1	A	12	12	7204	7215
	5	p1	A	10	10	8114	8123
	6	p1	A	12	12	8355	8366
	7	p1	A	13	13	8660	8672
	8	p4	ATAA	3	12	9320	9331
	9	p1	T	10	10	9474	9483
	10	p1	T	10	10	10382	10391
	11	p2	AT	6	12	10594	10605
	12	p2	AT	6	12	10611	10622
	13	p1	A	10	10	10817	10826
	14	p1	T	12	12	13712	13723
	15	p1	T	16	16	15634	15649
	84	p1	A	11	11	144186	144196
L. siceraria	1	p1	T	12	12	1526	1537
	2	p1	A	14	14	3225	3238
	3	p5	GACTT	3	15	3951	3965
	4	p2	TA	5	10	4565	4574
	5	p3	TAA	4	12	4647	4658
	6	p1	C	10	10	5427	5436
	7	p1	A	12	12	5589	5600
	8	p1	A	10	10	7397	7406
	9	p3	ATA	4	12	7621	7632
	10	p1	A	14	14	8614	8627
	11	p4	ATAA	3	12	9267	9278
	12	p2	AT	5	10	10539	10548
	13	p1	A	10	10	10703	10712
	14	p1	A	11	11	12914	12924
	15	p1	T	10	10	13632	13641
	16	p1	T	10	10	14322	14331
	17	p1	T	13	13	19693	19705
	18	p2	AT	5	10	21072	21081
	19	p4	AAAT	3	12	23863	23874
	20	p1	T	10	10	27401	27410
	21	p2	AT	5	10	28600	28609
	22	p2	TA	6	12	28611	28622
	23	p1	A	10	10	28719	28728
	24	p1	A	10	10	30048	30057

16	p1	T	13	13	19768	19780
17	p2	AT	5	10	21147	21156
18	p4	AAAT	3	12	23939	23950
19	p1	T	11	11	24228	24238
20	p2	TA	6	12	28695	28706
21	p1	A	10	10	28782	28791
22	p1	T	10	10	30077	30086
23	p4	TTTA	3	12	32836	32847
24	p1	T	11	11	33697	33707
25	p2	TA	6	12	38239	38250
26	p4	TAAT	3	12	38606	38617
27	p5	ATTTG	3	15	44296	44310
28	p1	A	13	13	44477	44489
29	p1	A	10	10	45868	45877
30	p1	A	12	12	46353	46364
31	p1	T	11	11	48852	48862
32	p1	T	10	10	57104	57113
33	p1	T	12	12	61896	61907
34	p1	T	14	14	65861	65874
35	p1	T	10	10	72761	72770
36	p1	A	15	15	73128	73142
37	p1	T	13	13	73901	73913
38	p1	T	13	13	82517	82529
39	p1	A	14	14	83533	83546
40	p1	T	10	10	84936	84945
41	p4	ATTT	4	16	90692	90707

25	p3	TTA	4	12	30222	30233
26	p4	TTTA	3	12	32864	32875
27	p2	AT	5	10	38382	38391
28	p1	A	10	10	38508	38517
29	p4	TAAT	3	12	38739	38750
30	p1	A	10	10	44596	44605
31	p1	A	11	11	46501	46511
32	p2	AT	5	10	49125	49134
33	p1	A	10	10	49325	49334
34	p1	T	10	10	51802	51811
35	p1	A	10	10	57274	57283
36	p1	T	10	10	59463	59472
37	p1	T	10	10	61945	61954
38	p1	T	12	12	65888	65899
39	p1	T	14	14	67416	67429
40	p1	T	10	10	69247	69256
41	p1	A	10	10	70740	70749
42	p1	A	10	10	73088	73097
43	p1	A	13	13	73195	73207
44	p1	T	10	10	73958	73967
45	p1	A	11	11	83623	83633
46	p1	T	10	10	83648	83657
47	p1	T	15	15	85000	85014
48	p1	T	10	10	86543	86552
49	p4	ATTT	4	16	90766	90781
50	p1	A	12	12	110511	110522

	42	p1	A	10	10	110450	110459
	43	p2	AG	5	10	110546	110555
	44	p4	AAAT	3	12	112953	112964
	45	p1	A	10	10	113203	113212
	46	p4	AAGT	3	12	113808	113819
	47	p3	TTA	4	12	115432	115443
	48	p1	T	12	12	115612	115623
	49	p2	AT	6	12	115638	115649
	50	p1	T	10	10	116070	116079
	51	p2	TA	5	10	116354	116363
	52	p1	T	11	11	116997	117007
	53	p4	CAAA	3	12	121696	121707
	54	p1	A	10	10	123157	123166
	55	p1	A	11	11	123248	123258
	56	p1	T	10	10	123320	123329
	57	p2	TC	5	10	123530	123539
	58	p1	T	10	10	127610	127619
	59	p1	A	11	11	129872	129882
	60	p2	CT	5	10	133197	133206
	61	p1	T	10	10	133293	133302
	62	p4	AAAT	4	16	153045	153060
C. grandis	1	p1	A	10	10	147	156
	2	p1	T	10	10	1539	1548
	3	p1	A	11	11	3229	3239
	51	p2	AG	5	10	110609	110618
	52	p4	AAAT	3	12	113015	113026
	53	p4	AAGT	3	12	113870	113881
	54	p3	TTA	4	12	115496	115507
	55	p2	TA	6	12	115700	115711
	56	p2	TA	5	10	116428	116437
	57	p1	T	11	11	117074	117084
	58	p4	CAAA	3	12	121789	121800
	59	p1	A	10	10	123342	123351
	60	p1	T	10	10	123491	123500
	61	p1	T	10	10	128798	128807
	62	p2	CT	5	10	133371	133380
	63	p1	T	12	12	133467	133478
	64	p4	AAAT	4	16	153208	153223
C. laevigata	1	p1	A	17	17	1778	1794
	2	p2	TA	6	12	1808	1819
	3	p1	A	17	17	3778	3794
	4	p1	A	11	11	3952	3962
	5	p1	A	10	10	4455	4464
	6	p1	A	10	10	4620	4629
	7	p1	A	12	12	4724	4735
	8	p3	CTT	4	12	4738	4749
	9	p1	A	11	11	4951	4961
	10	p4	TTTA	3	12	5003	5014

4	p5	GACTT	3	15	3956	3970
5	p1	A	10	10	4297	4306
6	p2	TA	5	10	4573	4582
7	p3	TAA	4	12	4655	4666
8	p1	A	13	13	5337	5349
9	p1	A	12	12	6488	6499
10	p1	A	10	10	7135	7144
11	p1	A	12	12	8350	8361
12	p1	A	10	10	10523	10532
13	p1	A	10	10	12729	12738
14	p1	T	11	11	13446	13456
15	p1	T	13	13	19503	19515
16	p2	AT	5	10	20882	20891
17	p1	T	10	10	27150	27159
18	p1	T	16	16	28175	28190
19	p4	TTTA	3	12	28439	28450
20	p1	T	11	11	29173	29183
21	p1	A	11	11	32164	32174
22	p4	TTTA	3	12	32711	32722
23	p1	A	11	11	32836	32846
24	p1	A	11	11	33960	33970
25	p4	ATTA	3	12	38609	38620
26	p1	A	16	16	44472	44487
27	p1	T	10	10	44798	44807
28	p1	T	10	10	47471	47480
29	p1	T	12	12	48824	48835

11	p1	T	10	10	5041	5050
12	p1	A	13	13	5211	5223
13	p4	TCAA	3	12	5365	5376
14	p1	T	10	10	6446	6455
15	p1	T	10	10	9024	9033
16	p1	A	10	10	9036	9045
17	p2	TA	5	10	9470	9479
18	p1	A	11	11	9816	9826
19	p3	TTC	4	12	9915	9926
20	p2	AT	7	14	11307	11320
21	p1	T	10	10	11550	11559
22	p1	T	13	13	11567	11579
23	p1	A	12	12	11596	11607
24	p2	AT	6	12	11610	11621
25	p1	A	10	10	11902	11911
26	p1	A	10	10	13492	13501
27	p1	A	15	15	14429	14443
28	p1	T	13	13	15011	15023
29	p1	A	10	10	15316	15325
30	p1	T	10	10	15780	15789
31	p3	TTA	5	15	16219	16233
32	p1	T	15	15	16494	16508
33	p1	A	13	13	17605	17617
34	p1	T	14	14	18367	18380
35	p1	A	15	15	18384	18398
36	p3	TTA	4	12	18527	18538

30	p1	A	10	10	49689	49698
31	p2	TA	9	18	49863	49880
32	p1	T	11	11	57124	57134
33	p1	T	10	10	57203	57212
34	p1	A	11	11	57253	57263
35	p1	A	11	11	59400	59410
36	p1	T	10	10	59461	59470
37	p1	T	11	11	61936	61946
38	p1	T	11	11	65888	65898
39	p1	T	11	11	67408	67418
40	p1	A	10	10	71465	71474
41	p1	A	13	13	73137	73149
42	p1	A	10	10	77812	77821
43	p1	T	11	11	82504	82514
44	p1	A	11	11	83519	83529
45	p1	T	16	16	83569	83584
46	p4	ATTT	4	16	90671	90686
47	p1	A	12	12	110412	110423
48	p2	AG	5	10	110510	110519
49	p1	A	10	10	113167	113176
50	p4	AAGT	3	12	113772	113783
51	p1	T	11	11	115567	115577
52	p2	TA	7	14	115591	115604
53	p2	TA	6	12	116307	116318
54	p2	AT	5	10	119756	119765

37	p3	TTA	4	12	18549	18560
38	p5	CAAAT	3	15	18562	18576
39	p1	T	13	13	20639	20651
40	p2	AT	5	10	21976	21985
41	p4	AAAG	3	12	25039	25050
42	p1	T	10	10	28299	28308
43	p3	TAT	4	12	30676	30687
44	p2	TA	5	10	30713	30722
45	p1	T	12	12	31401	31412
46	p6	GTAAG<br>A	3	18	31520	31537
47	p1	T	11	11	32060	32070
48	p1	T	11	11	32322	32332
49	p1	T	11	11	32337	32347
50	p4	CAAA	3	12	32455	32466
51	p2	AT	9	18	32488	32505
52	p1	A	12	12	32916	32927
53	p1	A	10	10	33020	33029
54	p2	TA	5	10	38858	38867
55	p1	A	15	15	39345	39359
56	p5	TTTAA	3	15	39858	39872
57	p1	T	13	13	46785	46797
58	p1	A	17	17	47763	47779
59	p1	A	11	11	48497	48507
60	p4	TTTA	3	12	50411	50422
61	p1	T	10	10	50426	50435

55	p4	TTGA	3	12	120352	120363
56	p4	CAAA	3	12	121663	121674
57	p1	A	10	10	123120	123129
58	p1	A	10	10	123211	123220
59	p2	AT	5	10	128088	128097
60	p1	T	10	10	128688	128697
61	p1	A	11	11	129935	129945
62	p1	T	12	12	130706	130717
63	p2	CT	5	10	133266	133275
64	p1	T	12	12	133362	133373
65	p4	AAAT	4	16	153099	153114

62	p3	ATA	4	12	50849	50860
63	p1	A	10	10	51062	51071
64	p2	TA	7	14	51730	51743
65	p1	T	10	10	52122	52131
66	p4	TATT	3	12	52854	52865
67	p1	T	14	14	53078	53091
68	p1	T	11	11	54530	54540
69	p1	T	14	14	55609	55622
70	p1	T	16	16	59085	59100
71	p1	T	20	20	61327	61346
72	p3	AAT	4	12	63454	63465
73	p2	TA	5	10	63540	63549
74	p1	T	11	11	63903	63913
75	p1	T	17	17	65130	65146
76	p1	T	19	19	67976	67994
77	p1	T	11	11	69328	69338
78	p1	T	13	13	69728	69740
79	p2	AT	6	12	69766	69777
80	p1	T	11	11	71609	71619
81	p1	A	11	11	72847	72857
82	p1	T	15	15	72881	72895
83	p1	T	10	10	73556	73565
84	p1	T	12	12	75001	75012
85	p1	A	13	13	75031	75043
86	p1	A	10	10	75206	75215
87	p1	A	12	12	76321	76332

88	p1	T	14	14	79701	79714
89	p1	T	12	12	82443	82454
90	p1	T	16	16	84040	84055
91	p1	T	15	15	84558	84572
92	p1	T	16	16	85549	85564
93	p1	A	12	12	85595	85606
94	p1	A	11	11	85622	85632
95	p1	T	10	10	85659	85668
96	p1	T	16	16	86354	86369
97	p1	T	10	10	86454	86463
98	p1	T	10	10	87209	87218
99	p1	T	15	15	88926	88940
100	p1	T	17	17	101699	101715
101	p1	T	11	11	103362	103372
102	p1	A	13	13	112229	112241
103	p2	AG	5	10	112342	112351
104	p1	A	10	10	115617	115626
105	p1	T	17	17	117331	117347
106	p4	AATA	3	12	117477	117488
107	p1	A	13	13	118014	118026
108	p1	A	11	11	118032	118042
109	p1	A	10	10	118163	118172
110	p1	T	10	10	118185	118194
111	p1	T	11	11	118943	118953
112	p4	AATA	3	12	120763	120774
113	p1	T	18	18	125734	125751

114	p1	A	13	13	126362	126374
115	p1	T	16	16	128568	128583
116	p1	T	10	10	129399	129408
117	p2	AT	5	10	130471	130480
118	p1	T	13	13	131732	131744
119	p3	TCC	4	12	132140	132151
120	p1	A	16	16	132262	132277
121	p2	CT	5	10	135714	135723
122	p1	T	13	13	135824	135836
123	p1	A	11	11	144693	144703
124	p1	A	17	17	146350	146366
125	p1	A	15	15	159125	159139

Table S7. Summary of Three categories of repeats and Simple Sequence Repeats (SSRs).

	Three categories of repeats				Simple Sequence Repeats (SSRs)						
**Species**	**Tandem repeat**	**Dispersed repeats**	**Palindromic repeats**	**Total**	**Mononucleotides**	**Dinucleotides**	**Trinucleotides**	**Tetranucleotides**	**Pentanucleotides**	**Hexnucleotides**	**Total**
G. cissiformis var. cissiformis	31	37	8	76	43	11	1	3	0	0	58
G. cissiformis var. villosa	17	29	13	59	45	11	2	5	0	0	63
H. lijiangensis	24	31	18	73	42	12	2	6	1	0	63
G. pentaphyllum	7	15	19	41	34	17	6	9	3	0	69
C. lanatus	6	11	18	35	39	11	2	9	1	0	62
C. grandis	9	16	11	36	46	9	1	8	1	0	65
C. sativus	19	21	29	69	55	10	5	10	4	4	88
C. moschata	9	26	23	58	53	10	2	6	0	1	72
M. charantia	13	24	23	60	65	5	3	6	5	0	84
L. siceraria	6	14	20	40	39	11	4	9	1	0	64
C. laevigata	22	23	26	71	91	14	9	8	2	1	125
**Sum**	163	247	208	618	552	121	37	79	18	6	813
**Persentage**	0.2638	0.3997	0.3366		0.6790	0.1488	0.0455	0.0972	0.0221	0.0074	

TableS8. Results of selective events analysis in kaks-calculator.

Genes	Method	Ka	Ks	Ka/Ks	P-Value(Fisher)	Length
accD	MA	0.0391047	0.0970002	0.403141	2.20E-13	7266
atpA	MA	0.0183613	0.106668	0.172134	1.15E-44	7602
atpB	MA	0.0110111	0.095761	0.114985	3.88E-53	7470
atpE	MA	0.0603167	0.051829	1.16376	0.559935	2016
atpH	MA	0.00363092	0.0356277	0.101913	7.52E-06	1215
atpI	MA	0.00904498	0.106914	0.0846003	2.70E-28	3705
cemA	MA	0.0135667	0.0894947	0.151592	3.50E-18	3417
clpP	MA	0.0399005	0.0731351	0.545572	0.00172801	2931
matK	MA	0.0557728	0.124893	0.446566	1.23E-16	7461
ndhA	MA	0.0252328	0.169295	0.149046	5.69E-55	5436
ndhB	MA	0.0028576	0.0165509	0.172655	4.44E-09	7650
ndhC	MA	0.0108038	0.103853	0.10403	1.32E-14	1800
ndhE	MA	0.010955	0.0795825	0.137656	1.79E-08	1500
ndhF	MA	0.0445693	0.251166	0.17745	4.97E-123	11346
ndhG	MA	0.0225714	0.0912776	0.247283	5.05E-12	2640
ndhH	MA	0.012389	0.157823	0.0784993	6.07E-69	5895
ndhI	MA	0.0179061	0.105445	0.169815	9.08E-15	2469
ndhJ	MA	0.00519531	0.081195	0.0639856	1.05E-19	2370
ndhK	MA	0.0123671	0.0659584	0.187499	6.46E-13	3390
petA	MA	0.017724	0.117075	0.151391	3.47E-36	4791
petB	MA	0.00187322	0.0943109	0.0198622	1.71E-30	2748
petD	MA	0.00486226	0.0657301	0.0739731	3.30E-15	2337
petG	MA	0.0015877	0.250091	0.0063485	0	555
petL	MA	0.0144454	0.0635308	0.227376	0.00181413	465
psaA	MA	0.00743671	0.0784927	0.0947439	4.40E-72	11250
psaB	MA	0.00364328	0.108354	0.0336239	1.71E-107	11010
psaC	MA	0.0010974	0.0738772	0.0148543	0	1215
psaI	MA	0.0244549	0.0490947	0.498117	0.126012	546
psaJ	MA	0.0871484	0.237348	0.367176	3.21E-06	651
psbA	MA	0.0012007	0.0497393	0.0241398	5.74E-30	5295
psbB	MA	0.0104063	0.116348	0.0894414	2.52E-70	7593
psbC	MA	0.00514963	0.116187	0.044322	1.48E-77	7023
psbD	MA	0.0018744	0.0904334	0.0207269	5.31E-50	5295
psbE	MA	0.00502008	0.0154544	0.324833	0.0432207	1245
psbF	MA	0.0045827	0.106617	0.0429827	4.76E-08	585
psbH	MA	0.0305251	0.129638	0.235463	6.66E-07	1095

psbI	MA	0.000330199	0.330199	0.001	0	540
psbJ	MA	0.0292206	0.190266	0.153578	8.89E-09	600
psbK	MA	0.0134422	0.19685	0.0682864	1.13E-13	915
psbL	MA	0.0138964	0.0224061	0.620205	0.48334	570
psbM	MA	5.90E-05	0.0589522	0.001	0	510
psbT	MA	0.0318224	0.0780977	0.407468	0.0268681	534
psbZ	MA	0.0193121	0.0422036	0.457593	0.0624333	930
rbcL	MA	0.0156003	0.128713	0.121202	2.37E-63	7119
rpl2	MA	0.00222206	0.0164971	0.134694	6.20E-06	4113
rpl14	MA	0.00187043	0.0564866	0.0331128	3.04E-13	1830
rpl16	MA	0.0215481	0.170325	0.126511	2.38E-20	1881
rpl22	MA	0.0547057	0.148496	0.368399	9.77E-10	2475
rpl23	MA	0.00457018	0.0136748	0.334204	0.0324472	1395
rpl32	MA	0.165708	0.353357	0.468953	1.19E-05	780
rpl33	MA	0.0139895	0.125399	0.11156	2.93E-09	987
rpl36	MA	0.121751	0.353626	0.344293	2.60E-07	534
rpoA	MA	0.017816	0.100333	0.177569	4.91E-25	4953
rpoB	MA	0.00712916	0.0924444	0.0771184	2.14E-116	16050
rpoC1	MA	0.00659646	0.08053	0.081913	3.82E-66	10200
rpoC2	MA	0.0242062	0.105972	0.22842	2.36E-95	20820
rps2	MA	0.00775838	0.0972066	0.0798133	3.07E-28	3540
rps3	MA	0.0222735	0.165873	0.134281	4.31E-34	3270
rps4	MA	0.020759	0.0509954	0.407075	0.000401329	3015
rps7	MA	0.00156787	0.00686606	0.228351	0.018672	2325
rps8	MA	0.01752	0.217991	0.0803704	2.00E-31	2010
rps11	MA	0.00538826	0.128837	0.0418224	8.49E-29	2070
rps14	MA	0.011867	0.0544262	0.218038	7.88E-06	1500
rps15	MA	0.0355231	0.167431	0.212166	3.44E-09	1350
rps18	MA	0.017931	0.0276772	0.64786	0.2408	1515
rps19	MA	0.015629	0.4315	0.0362202	1.43E-38	993
ycf3	MA	0.00264531	0.0800349	0.033052	9.47E-22	2520
ycf4	MA	0.02458	0.100557	0.244439	4.80E-13	2736

Table S9. Codon usage and relative synonymous codon usage (RSCU) value for protein-coding genes in the ten Cucurbitaceae choroplast genomes.

		G.cissiformis var. cissiformis		*G.cissiformis var. villosa*		*H.lijiangensis*		*G. pentaphyllum*		*C.lanatus*		*C.grandis*		*C.sativus*		*C. moschata*		*L. siceraria*		*M. charantia*		*C.laevigata*	
**Amino Acid**	**Codon**	**Number**	**RSCU**	**Number**	**RSCU**	**Number**	**RSCU**	**Number**	**RSCU**	**Number**	**RSCU**	**Number**	**RSCU**	**Number**	**RSCU**	**Number**	**RSCU**	**Number**	**RSCU**	**Number**	**RSCU**	**Number**	**RSCU**
**Phe**	UUU	1013	1.31	1011	1.31	993	1.3	997	1.3	963	1.29	984	1.3	942	1.29	933	1.28	962	1.29	993	1.31	903	1.26
**Phe**	UUC	534	0.69	534	0.69	538	0.7	533	0.7	526	0.71	533	0.7	523	0.71	530	0.72	525	0.71	524	0.69	527	0.74
**Leu**	UUA	883	1.9	884	1.89	892	1.9	893	1.89	862	1.89	875	1.88	871	1.91	853	1.87	857	1.87	851	1.88	811	1.82
**Leu**	UUG	558	1.2	563	1.21	561	1.19	574	1.22	555	1.21	566	1.22	541	1.19	563	1.24	563	1.23	547	1.21	565	1.27
**Leu**	CUU	592	1.27	592	1.27	600	1.27	593	1.26	574	1.26	591	1.27	575	1.26	570	1.25	568	1.24	569	1.26	560	1.25
**Leu**	CUC	206	0.44	205	0.44	197	0.42	209	0.44	195	0.43	194	0.42	188	0.41	182	0.4	197	0.43	193	0.43	185	0.41
**Leu**	CUA	373	0.8	377	0.81	378	0.8	375	0.8	377	0.82	383	0.82	391	0.86	381	0.84	380	0.83	378	0.84	381	0.85
**Leu**	CUG	179	0.38	179	0.38	196	0.42	184	0.39	180	0.39	183	0.39	172	0.38	181	0.4	187	0.41	178	0.39	177	0.4
**Ile**	AUU	1111	1.45	1113	1.45	1113	1.45	1112	1.44	1077	1.46	1112	1.46	1097	1.46	1088	1.45	1097	1.46	1082	1.44	1089	1.47
**Ile**	AUC	459	0.6	458	0.6	457	0.6	464	0.6	466	0.63	466	0.61	454	0.61	471	0.63	472	0.63	449	0.6	440	0.59
**Ile**	AUA	733	0.95	728	0.95	730	0.95	742	0.96	667	0.91	705	0.93	698	0.93	687	0.92	682	0.91	717	0.96	699	0.94
**Met**	AUG	621	1	620	1	627	1	628	1	615	1	627	1	605	1	615	1	616	1	614	1	588	1
**Val**	GUU	539	1.48	535	1.48	529	1.45	535	1.46	511	1.44	521	1.43	503	1.43	512	1.47	513	1.44	513	1.45	498	1.41
**Val**	GUC	177	0.49	178	0.49	186	0.51	178	0.49	193	0.55	193	0.53	196	0.56	184	0.53	189	0.53	179	0.5	173	0.49
**Val**	GUA	541	1.49	541	1.49	539	1.48	548	1.5	520	1.47	548	1.51	512	1.46	509	1.46	536	1.5	532	1.5	542	1.54
**Val**	GUG	195	0.54	195	0.54	207	0.57	200	0.55	191	0.54	194	0.53	196	0.56	192	0.55	190	0.53	195	0.55	197	0.56
**Ser**	UCU	573	1.66	578	1.66	575	1.65	573	1.66	572	1.69	572	1.67	577	1.72	566	1.67	571	1.67	561	1.67	548	1.64
**Ser**	UCC	356	1.03	357	1.03	350	1.01	336	0.97	329	0.97	328	0.96	305	0.91	324	0.96	332	0.97	324	0.96	331	0.99
**Ser**	UCA	415	1.2	420	1.21	427	1.23	426	1.23	426	1.26	429	1.25	412	1.23	421	1.24	424	1.24	425	1.26	414	1.24
**Ser**	UCG	193	0.56	193	0.56	195	0.56	204	0.59	190	0.56	197	0.57	196	0.59	192	0.57	199	0.58	187	0.56	188	0.56

**Pro**	CCU	422	1.53	421	1.53	410	1.49	419	1.52	419	1.55	425	1.56	430	1.56	411	1.53	414	1.53	409	1.54	421	1.58
**Pro**	CCC	230	0.84	230	0.83	221	0.8	227	0.82	200	0.74	204	0.75	207	0.75	196	0.73	205	0.76	204	0.77	201	0.75
**Pro**	CCA	307	1.12	309	1.12	329	1.19	306	1.11	310	1.15	305	1.12	323	1.17	315	1.17	315	1.17	313	1.18	313	1.17
**Pro**	CCG	141	0.51	143	0.52	144	0.52	149	0.54	149	0.55	158	0.58	145	0.52	156	0.58	146	0.54	137	0.52	132	0.49
**Thr**	ACU	555	1.64	553	1.63	547	1.61	541	1.57	537	1.62	549	1.61	569	1.68	523	1.62	536	1.62	537	1.63	523	1.63
**Thr**	ACC	238	0.7	242	0.71	254	0.75	256	0.75	251	0.76	252	0.74	248	0.73	237	0.73	247	0.75	239	0.73	227	0.71
**Thr**	ACA	410	1.21	410	1.21	403	1.19	421	1.23	391	1.18	409	1.2	397	1.17	384	1.19	395	1.2	388	1.18	404	1.26
**Thr**	ACG	153	0.45	154	0.45	153	0.45	156	0.45	144	0.44	150	0.44	140	0.41	147	0.46	144	0.44	150	0.46	132	0.41
**Ala**	GCU	633	1.82	631	1.82	639	1.82	637	1.84	630	1.84	640	1.82	618	1.79	622	1.81	628	1.82	628	1.85	648	1.89
**Ala**	GCC	234	0.67	230	0.66	231	0.66	224	0.65	218	0.64	226	0.64	227	0.66	222	0.65	227	0.66	216	0.64	202	0.59
**Ala**	GCA	380	1.09	381	1.1	385	1.1	374	1.08	374	1.09	392	1.11	388	1.12	379	1.1	380	1.1	371	1.1	385	1.12
**Ala**	GCG	148	0.42	148	0.43	148	0.42	148	0.43	149	0.43	150	0.43	148	0.43	153	0.44	146	0.42	140	0.41	135	0.39
**Tyr**	UAU	787	1.59	793	1.59	793	1.58	793	1.58	774	1.61	796	1.6	779	1.58	769	1.61	778	1.6	789	1.61	767	1.6
**Tyr**	UAC	203	0.41	203	0.41	211	0.42	211	0.42	190	0.39	197	0.4	206	0.42	189	0.39	195	0.4	194	0.39	189	0.4
**TER**	UAA	55	1.88	58	2	57	1.97	56	1.93	54	1.91	54	1.91	51	1.8	53	1.87	56	1.95	56	1.98	50	1.81
**TER**	UAG	15	0.51	14	0.48	14	0.48	15	0.52	16	0.56	16	0.56	15	0.53	16	0.56	16	0.56	14	0.49	19	0.69
**His**	CAU	490	1.52	486	1.52	495	1.53	493	1.53	469	1.52	480	1.52	449	1.51	468	1.51	476	1.52	468	1.52	491	1.55
**His**	CAC	153	0.48	153	0.48	151	0.47	151	0.47	149	0.48	150	0.48	145	0.49	151	0.49	151	0.48	147	0.48	143	0.45
**Gln**	CAA	724	1.54	722	1.54	729	1.54	735	1.55	718	1.54	724	1.54	694	1.53	719	1.54	727	1.55	716	1.54	705	1.55
**Gln**	CAG	215	0.46	216	0.46	219	0.46	211	0.45	213	0.46	218	0.46	216	0.47	214	0.46	214	0.45	216	0.46	206	0.45
**Asn**	AAU	1022	1.56	1028	1.56	1021	1.56	1001	1.54	985	1.54	1018	1.55	984	1.55	979	1.53	990	1.54	980	1.54	987	1.55
**Asn**	AAC	291	0.44	294	0.44	287	0.44	301	0.46	291	0.46	294	0.45	282	0.45	301	0.47	292	0.46	290	0.46	287	0.45
**Lys**	AAA	1085	1.51	1089	1.51	1098	1.5	1096	1.5	1023	1.51	1070	1.5	1032	1.48	1014	1.5	1040	1.5	1045	1.51	1028	1.49
**Lys**	AAG	351	0.49	350	0.49	365	0.5	367	0.5	333	0.49	353	0.5	363	0.52	337	0.5	349	0.5	338	0.49	349	0.51

**Asp**	GAU	867	1.6	869	1.61	868	1.62	884	1.62	864	1.61	874	1.61	832	1.6	891	1.6	869	1.6	860	1.62	871	1.63
**Asp**	GAC	214	0.4	212	0.39	206	0.38	209	0.38	212	0.39	212	0.39	210	0.4	225	0.4	218	0.4	204	0.38	198	0.37
**Glu**	GAA	1046	1.49	1043	1.49	1041	1.49	1045	1.49	1028	1.49	1061	1.5	991	1.51	1024	1.5	1030	1.49	1037	1.49	1010	1.49
**Glu**	GAG	357	0.51	358	0.51	355	0.51	354	0.51	348	0.51	351	0.5	322	0.49	344	0.5	353	0.51	355	0.51	346	0.51
**Cys**	UGU	226	1.48	224	1.48	225	1.48	232	1.49	208	1.45	215	1.45	208	1.43	212	1.44	212	1.46	218	1.47	228	1.52
**Cys**	UGC	79	0.52	79	0.52	80	0.52	80	0.51	78	0.55	81	0.55	82	0.57	83	0.56	78	0.54	78	0.53	72	0.48
**TER**	UGA	18	0.61	15	0.52	16	0.55	16	0.55	15	0.53	15	0.53	19	0.67	16	0.56	14	0.49	15	0.53	14	0.51
**Trp**	UGG	475	1	473	1	478	1	473	1	464	1	469	1	445	1	455	1	464	1	461	1	455	1
**Arg**	CGU	353	1.32	353	1.31	352	1.32	353	1.32	340	1.32	351	1.33	333	1.32	340	1.33	349	1.33	342	1.33	356	1.36
**Arg**	CGC	107	0.4	105	0.39	107	0.4	105	0.39	95	0.37	97	0.37	93	0.37	100	0.39	94	0.36	103	0.4	98	0.37
**Arg**	CGA	389	1.45	388	1.45	387	1.45	378	1.41	375	1.45	387	1.46	374	1.48	352	1.38	373	1.42	362	1.4	356	1.36
**Arg**	CGG	95	0.35	94	0.35	103	0.39	108	0.4	116	0.45	112	0.42	110	0.44	114	0.45	117	0.45	105	0.41	105	0.4
**Ser**	AGU	415	1.2	414	1.19	411	1.18	405	1.17	388	1.15	404	1.18	391	1.17	405	1.2	397	1.16	388	1.15	410	1.23
**Ser**	AGC	124	0.36	121	0.35	128	0.37	130	0.38	128	0.38	129	0.38	126	0.38	122	0.36	127	0.37	134	0.4	113	0.34
**Arg**	AGA	488	1.82	493	1.84	484	1.81	493	1.84	459	1.78	480	1.81	450	1.78	458	1.79	475	1.81	474	1.84	488	1.87
**Arg**	AGG	178	0.66	178	0.66	169	0.63	168	0.63	163	0.63	162	0.61	155	0.61	168	0.66	167	0.64	161	0.62	166	0.63
**Gly**	GGU	603	1.34	604	1.34	598	1.32	610	1.35	593	1.35	601	1.33	602	1.36	591	1.34	602	1.35	588	1.35	594	1.37
**Gly**	GGC	174	0.39	173	0.38	165	0.36	174	0.39	171	0.39	170	0.38	172	0.39	174	0.4	167	0.37	165	0.38	159	0.37
**Gly**	GGA	742	1.65	747	1.65	750	1.66	726	1.61	721	1.64	738	1.64	706	1.59	717	1.63	727	1.63	707	1.63	715	1.64
**Gly**	GGG	283	0.63	284	0.63	296	0.65	293	0.65	274	0.62	294	0.65	291	0.66	278	0.63	291	0.65	276	0.64	271	0.62
**Total**		**26726**		**26746**		**26813**		**26828**		**26016**		**26634**		**25922**		**25978**		**26251**		**26029**		**25785**	
**>1**			31		31		31		30		30		30		30		30		30		30		30
**=1**			2		2		2		2		2		2		2		2		2		2		2
**<1**			31		31		31		32		32		32		32		32		32		32		32

Table S10. Base compositions for protein-coding genes in the ten Cucurbitaceae chloroplast genomes.

**Species**	T3s	C3s	A3s	G3s	CAI	CBI	Fop	Nc	GC3s	GC	L_sym	L_aa
G.cissiformis var. cissiformis	0.4687	0.1736	0.4331	0.1755	0.165	-0.103	0.352	49.56	0.267	0.377	25542	26638
G.cissiformis var. villosa	0.4684	0.1732	0.4335	0.1758	0.165	-0.104	0.352	49.53	0.267	0.377	25566	26659
H.lijiangensis	0.4662	0.1728	0.434	0.1783	0.165	-0.104	0.352	49.7	0.269	0.378	25621	26726
G. pentaphyllum	0.4662	0.1735	0.4332	0.1787	0.165	-0.103	0.352	49.72	0.269	0.377	25640	26741
C.lanatus	0.4674	0.1742	0.431	0.1775	0.167	-0.099	0.355	49.73	0.269	0.379	24852	25931
C.sativus	0.4671	0.1731	0.4303	0.1773	0.166	-0.101	0.353	49.77	0.269	0.378	24787	25837
C.grandis	0.4675	0.1719	0.4336	0.1781	0.166	-0.102	0.353	49.66	0.268	0.378	25453	26549
C. moschata	0.4667	0.1743	0.4302	0.1804	0.167	-0.1	0.355	49.94	0.271	0.38	24823	25893
M. charantia	0.469	0.1721	0.4348	0.1769	0.167	-0.103	0.353	49.59	0.267	0.376	24869	25944
L. siceraria	0.4661	0.1739	0.4313	0.1794	0.167	-0.102	0.353	49.79	0.27	0.38	25085	26165
C.laevigata	0.4708	0.1687	0.4344	0.1771	0.166	-0.107	0.35	49.23	0.264	0.378	24659	25702

Notes: GC : GC content; GC3s: GC Content.of the Third Codon Base; CAI: Codon Adaptability Index; CBI: Codon Bias Index; FOP: Optimal Codon Usage Frequency; L_sym: Number of Synonymous Codons; L_aa: Total Number of Amino Acid.
